# Interactome Analysis Reveals a Link of the Novel ALMS1-CEP70 Complex to Centrosomal Clusters

**DOI:** 10.1016/j.mcpro.2023.100701

**Published:** 2023-12-18

**Authors:** Franziska Woerz, Felix Hoffmann, Shibu Antony, Sylvia Bolz, Mohamed Ali Jarboui, Katrin Junger, Franziska Klose, Isabel F. Stehle, Karsten Boldt, Marius Ueffing, Tina Beyer

**Affiliations:** Eberhard Karls University Tübingen, Institute for Ophthalmic Research, University of Tübingen, Tübingen, Germany

**Keywords:** Alström syndrome, cilia, centrosome;, basal body, ciliopathy, protein complex analysis, CRISPR/Cas9

## Abstract

Alström syndrome (ALMS) is a very rare autosomal-recessive disorder, causing a broad range of clinical defects most notably retinal degeneration, type 2 diabetes, and truncal obesity. The *ALMS1* gene encodes a complex and huge ∼0.5 MDa protein, which has hampered analysis in the past. The ALMS1 protein is localized to the centrioles and the basal body of cilia and is involved in signaling processes, for example, TGF-β signaling. However, the exact molecular function of ALMS1 at the basal body remains elusive and controversial. We recently demonstrated that protein complex analysis utilizing endogenously tagged cells provides an excellent tool to investigate protein interactions of ciliary proteins. Here, CRISPR/Cas9-mediated endogenously tagged ALMS1 cells were used for affinity-based protein complex analysis. Centrosomal and microtubule-associated proteins were identified, which are potential regulators of ALMS1 function, such as the centrosomal protein 70 kDa (CEP70). Candidate proteins were further investigated in ALMS1-deficient hTERT-RPE1 cells. Loss of ALMS1 led to shortened cilia with no change in structural protein localization, for example, acetylated and ɣ-tubulin, Centrin-3, or the novel interactor CEP70. Conversely, reduction of CEP70 resulted in decreased ALMS1 at the ciliary basal body. Complex analysis of CEP70 revealed domain-specific ALMS1 interaction involving the TPR-containing C-terminal (TRP-CT) fragment of CEP70. In addition to ALMS1, several ciliary proteins, including CEP135, were found to specifically bind to the TPR-CT domain. Data are available *via* ProteomeXchange with the identifier PXD046401. Protein interactors identified in this study provide candidate lists that help to understand ALMS1 and CEP70 function in cilia-related protein modification, cell death, and disease-related mechanisms.

## Highlights

• Definition of new ALMS1 interactors by affinity purification using endogenously tagged cells.
• The ALMS1 interactor CEP70 stabilizes ALMS1 at the basal body.
• Loss of CEP70 results in cell viability reduction independent of ALMS1.
• CEP70 protein complex reveals ciliary protein interaction.

## Graphical Abstract

**Figure:**
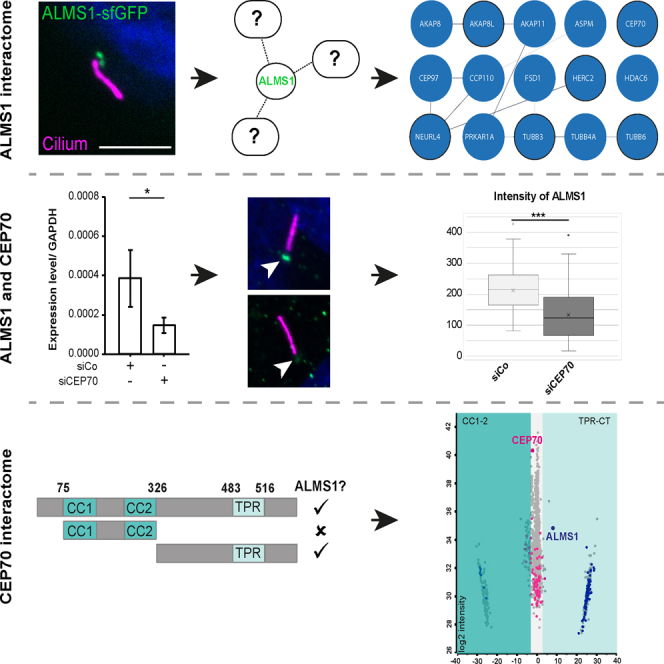

## In Brief

CRISPR/Cas9-mediated endogenously tagged ALMS1 HEK293T cells were used for affinity-based protein complex analysis. Centrosomal and microtubule-associated proteins were identified, which are potential regulators of ALMS1 function, such as CEP70. Loss of ALMS1 led to shortened cilia with no change in structural protein localization. Conversely, reduction of CEP70 resulted in decreased ALMS1 at the ciliary basal body. Complex analysis of CEP70 revealed ciliary proteins, including CEP135, which specifically bind to the TPR-CT domain.

Primary cilia are microtubule-based antenna-like structures, which can be found on nearly every cell in the human body. They appear, among others, as kinocilia, involved in the hearing process, or as connecting cilium in retinal photoreceptors, and are indispensable for vision (1). Overall, their sensory and signal-transducing functions are crucial for human health. Defects in cilia structure and function can lead to a wide range of pathologies in humans, also known as ciliopathies (2). To date, 35 ciliopathies are known, including the closely related “obesity ciliopathies” Bardet-Biedl syndrome (BBS, OMIM #209900) and Alström syndrome (ALMS, OMIM #203800). Both syndromes share similar phenotypic features, such as obesity, type 2 diabetes, and retinal dystrophy with polydactyly, although polydactyly only occurs in patients with BBS to our knowledge. Furthermore, BBS is a polygenic disease caused by 21 genes with a prevalence of 1:200,000, while ALMS is a monogenic ciliopathy affecting 1 to 9 out of 1 million individuals (3, 4, 5). The *ALMS1* gene is located on chromosome 2 (2p13.1) and exhibits 23 exons with three mutational hotspots in exons 8, 10, and 16. Over 300 mutations have been identified so far, whereby 49% of the mutations can be found in exon 8. Most of them are nonsense or frameshift mutations leading to a truncated ALMS1. However, the stability of the mRNA or any truncated protein product is unclear.

The *ALMS1* gene codes for an ubiquitously expressed protein, that associates with the proximal end of centrioles and the basal body of cilia (6). Primary cilia consist of a ciliary membrane, ciliary tip, microtubule backbone (axoneme), transition zone, and basal body (BB) (7). The BB is the basis of an emerging cilium, in mitotic cells called the centrosome (8). Centrosomes are known as the microtubule organization center (MTOC) and are crucial for cell signaling/transport processes, cell division, and motility. Its dysregulation has been implicated in various diseases, such as cancer and retinal degeneration (9). It is postulated, that the ∼460 kDa ALMS1 protein is important for cilia maintenance and function, endosomal trafficking, cell cycle control, metabolic homeostasis, and cell differentiation (4, 10), but the role of ALMS1 in ciliogenesis still needs to be elucidated. To date, some studies suggest an ALMS1 function in primary cilia formation and centrosomal cohesion (4, 11, 12, 13), whereas others describe an indirect role of ALMS1 in cilia assembly and disassembly (14, 15). Despite the published data, the precise molecular function of ALMS1 is still unclear.

In this study, we investigated the role of ALMS1 and associated proteins in ciliated cells. We hypothesized that ALMS1 interacts with centrosomal and ciliary proteins to regulate cilia stability and signaling. Therefore, CRISPR/Cas9 mediated endogenously tagged ALMS1 cells were used for affinity purification to identify interactors of full-length ALMS1. Results gave a first hint towards potential functional clusters in a cilium-related context. Moreover, ALMS1-deficient human retinal epithelial cells (hTERT-RPE1) were used to investigate ALMS1-relevant candidate proteins. Domain-specific protein complex analysis of the ALMS1 interactor and centrosomal protein CEP70 provides insights into ALMS1 and CEP70 clusters of centrosomal and cilia-related proteins. Functional validation hinted towards an upstream role of CEP70 for ALMS1 localization.

## Experimental Procedures

### Cell Lines and Cell Culture

HEK293T (CRL-3216, ATCC) and hTERT-RPE1 (CRL-4000, ATCC) were grown in Dulbecco’s Modified Eagle’s Medium–high glucose (DMEM, D6429, Sigma Aldrich and 41,966,029, Gibco) supplemented with 10% fetal bovine serum (FBS, F7524, S0615, Sigma Aldrich) and 0.5% Penicillin/Streptavidin (Pen/Strep, 15,140–122, Life Technologies) at 37 °C and 5% CO_2_. All cells were regularly tested for *mycoplasma*.

### Cloning of CRISPR/Cas9 Constructs

The pSpCas9(BB)-2A-Puro (PX459) V2.0 (was a gift from Feng Zhang, Addgene plasmid # 48139; http://n2t.net/addgene:62,988; RRID: Addgene_62988) was used for knockout (KO) and knockin (KI) experiments. SgRNA targeting ALMS1 exon 8 and 10 were designed using CCTop: An Intuitive, Flexible, and Reliable CRISPR/Cas9 Target Predicition Tool from Stemmer *et al*. (16). The sgRNA top and bottom oligonucleotides were phosphorylated and annealed. The PX459 vector was cut with the restriction enzyme Bbs1, followed by ligase-mediated insertion of the double-stranded sgRNA. The cloned PX459 was transformed *via* heat shock into competent *E. coli* DH5α (Library Efficiency DH5α Competent Cells, 18,263–012, Invitrogen) and plated on selective ampicillin (K029.1, Roth) agar plates. The final vector DNA was isolated using PureYield Plasmid Midi Preparation Kit (A2495, Promega) and sent for Sanger Sequencing (Eurofins, MicroSynth).

### Gateway Cloning

pEBTetBI-CLIP-Cep70 (gift from Kai Johnsson (Addgene plasmid #136870; http://n2t.net/addgene:136870;RRID:Addgene_136870) was used for Gateway cloning. Primers with gene-specific sequences of CEP70 with full-length attb1 and attb2 sequences were designed and purchased from IDT. PCRs and BP clonase (11789020, Invitrogen) recombination reactions were conducted according to manufacturer instruction. LR Reaction (11791020, Invitrogen) was performed to transfer the DNA fragments into Strep/FLAG destination vectors. DNA was isolated according to MonarchPlasmid Miniprep Kit (T1010L,New England BioLabs) and the PureYield Plasmid Midiprep Protocol System (A2495, Promega).

### Generation of CRISPR/Cas9 Mediated KOs in hTERT-RPE1 Cell Lines

Cells were transfected using Lipofectamine 3000 (L3000–015, Invitrogen). The PX459 vector without any sgRNA was used as control. Cells were transfected, followed by puromycin selection (A1113803, Thermo Fisher Scientific). For hTERT-RPE1 40 μg/ml puromycin were added for 5 days. After Puromycin selection a single clone selection was performed. The cells were seeded in a low concentration on a 14 cm Petri dish for three to 5 days, and colonies were transferred to new plates. Single clone expansion was followed by genomic DNA isolation using QuickDNA Extract Solution (101094, Biozym). The region of interest was amplified by PCR and send for sequencing (Eurofins, MicroSynth).

### Knockdown in hTERT-RPE1 Cells

HTERT-RPE1 native (wt), Cas control and ALMS1 KO were seeded with a confluency of approx. 70% for CEP70 siRNA transfection. A TriFECTa DsiRNA Kit (hs.Ri.CEP70.13) from IDT was used, including a negative control (DS NC1) and a pooled mixture of three siRNAs against CEP70 (hs.RI.CEP70.13.1-3). Lipofectamine 3000 (L3000-015, Invitrogen) was used according to the manufacturer protocol without P3000 for siRNA transfection.

### Real-Time qPCR

After transfection, cells were incubated for 24h followed by 48h of starvation. Total RNA from control and ALMS1 deficient cells were isolated using Trifast (30–2010, VWR), Chloroform (1.02445, Merck), Isopropanol (34,965, Honeywell) and 75% Ethanol (Ethanol absolute, 1.00983, Honeywell). CDNA was synthesized using reverse transcriptase (M1705, Promega), Hexamers (C1181, Promega), Oligo Nucleotide (C118A, Promega). SYBR Green master mix (1725274, BioRad) and BioRad qPCR Cycler was used to perform qPCR amplification and measurement. Primers and qPCR conditions are listed in the supplement table.

### Affinity Purification

Wildtype and endogenously ALMS1 tagged HEK293T cells were cultivated in DMEM supplements (described above) at 37 °C in 5% CO2. The cells were starved overnight before lysis. For Flag IP, overexpression constructs harboring N-terminal Strep Flag (NSF) -CEP70 and NSF-RAF1 were transfected with polyethylenimine solution (PEI, 23,966–2, PolyScience Inc, homemade) 48 h before lysis. All cells were grown in 14 cm dishes for affinity purification. Cells were lysed with lysis buffer (0.5% Nonidet-P40 (NP40, 11754599001, Roche); 2% protease inhibitor mixture (11 83 61 45 001, Roche) and 1% phosphatase inhibitor cocktail 2 and 3 (P5726, P0044, Sigma Aldrich), followed by a 30 min incubation in an end-over-end rotator (NeoLab) at 4 °C. The samples were centrifuged for 10 min at 10,000*g* at 4 °C. To measure the protein concentration of the supernatant, the Bradford Assay (500–0006, BioRad) was performed ensuring an equal amount of protein per experiment. Lysates were incubated with GFP (ChromoTek GFP-Trap Agarose, Proteintech) or Flag agarose beads (A2220, Sigma Aldrich) for 1 h at 4 °C. Lysate-bead mixtures were washed three times with washing buffer (0.1% Nonidet-P40, 1% phosphatase inhibitor cocktail 2 and 3 in TBS). For elution 0.2 M Glycine (pH 2.5) and 1M Tris base buffer (pH 10.4) was used for GFP-based affinity purification, followed by precipitation with LC-MS Methanol (1.06035, Merck) and chloroform (1.02445.1000, VWR). A subsequent protein digest was performed (DTT, 1.11474.0025, VWR; IAA 8.04744.0025, VWR; ABC, A6141, Sigma, and trypsin, T6567, Sigma; 37,286.04, Serva), which was inactivated with TFA (40967, Fluka). Lysat-Flag beads were washed three times with 1× TBS (1% phosphatase inhibitor cocktail 2 and 3), followed by an on-bead digest with urea buffer (2 M Urea, 50 mM Tris-HCl, pH 7.5) supplemented with trypsin and 1 mM DTT. IAA was added to the supernatant and incubated overnight at 37 °C. Trypsin was inactivated using TFA. Afterwards, the samples were purified using stop-and-go extraction tips (Sp301, StageTips, Thermo Scientific USA) and solutions containing 5% TFA in 80%, 50% Acetonitrile (ACN, 34,967-1L, Sigma) or HPLC water (1.153.33, Merck). The volume was reduced to max. 5 μl using a SpeedVac. 0.5%TFA was added to a final sample volume of 15 μl. Total 5 μl of this solution was injected for the mass spectrometry measurement.

### LC-MS/MS Analysis

An Ultimate3000 RSLCnano (Thermo Scientific) was coupled with a nanoelectrospray ion source to the Orbitrap Fusion Tribrid mass spectrometer (Thermo Scientific). The tryptic peptide mixtures were loaded onto a μPAC trapping column (C18 trapping column with pillar diameter of 5 μm, inter pillar distance of 2.5 μm pillar length/bed depth of 18 μm, external porosity of 9%, bed channel width of 2 mm and a length of 10 mm, porous shell thickness of 300 nm and pore size of 100–200 Å; Thermo Scientific, Germany). For injection the flow rate of 10 μl/min in 0.1% trifluoroacetic in HPLC grade water. The peptides were eluted after 3 min and separated on an analytical 50 cm μPAC C18 nano column (pillar diameter 5 μm, inter pillar distance 2.5 μm pillar length/bed depth 18 μm, external porosity of 59%, bed channel width of 315 μm and a length of 50 cm, porous shell thickness of 300 nm and pore size of 100–200 Å; Thermo Scientific/Dionex, Germany). The flow rate was at 300 nl/min over 120 min with a linear gradient from 2% to 30% of buffer B (80% acetonitrile and 0.08% formic acid in HPLC water) in buffer A (2% acetonitrile and 0.01% formic acid in HPLC water). A short gradient from 30% to 95% buffer B in 5 min enables the elution of remaining peptides. The subsequent analysis of the eluted peptides was performed on the Orbitrap Fusion Tribrid/Q Exactive mass spectrometer. The MaxQuant software (version 1.6.0.16, 1.6.1.0) was used to analyze the received MS/MS data. Trypsin/P was chosen as the digesting enzyme with maximal two missed cleavages. Methionine oxidation and N-terminal acetylation were selected for variable modifications. For fixed modifications, Cysteine carbamidomethylation was set. The data were analyzed by label-free quantification with the minimum ratio count of two and no fast LFQ. The re-quantify was chosen. The human SwissProt database (release 2021–05 for CEP70 PPI analysis (20,395 entries) and 2023 to 9 for ALMS1-GFP PPI analysis (20,422 entries)) was selected for peptide and protein identification. The contaminants were excluded using the MaxQuant contaminant search. A minimum peptide number of 1 and a minimum peptide length of 7 was tolerated.

### Experimental Design and Statistical Rationale

For quantification, unique and razor peptides were set. The Perseus software (version 1.6.14.0) was used for statistical analysis (17). Six biological replicates for each bait/tagged single clone and controls (RAF1 for CEP70, untagged cells for endogenously tagged ALMS1-sfGFP) are available for statistics. Potential contaminants and peptides only identified by side or reverse sequence were excluded for all data. Within the groups, a minimum half +1 of the samples should have valid values. Next, imputation by 0 was done for NaN values, the median values for each group followed by the ratio “specific bait/control” were calculated. Proteins which were significantly more abundant in one group were determined based on two statistical tests and classified in a two Tier system. Tier 1 interactors are stringently filtered with significance A (Benjamini-Hochberg FDR <0.05) and Student’s *t* test (Permutation-based FDR <0.05). Tier 2 includes additional proteins with a less stringent analysis (Significance A Benjamini Hochberg FDR <0.05 and Student’s *t* test *p*-value *p* < 0.05). The *t* test, two-sample test, was used to identify proteins, that were enriched stably within groups. For ALMS1-sfGFP data, three independent single clones were used for affinity purification. Tier 2 proteins which were present with minimum two clones are shown in the protein network (Fig. 3). Network was generated using experimental and functional interactions extracted from curated database using STRING (18) and visualized in Cytoscape 3.9.1 (https://cytoscape.org/) (19).

### Antibodies

The following antibodies were used for localization studies and Western blot: ALMS1 (1:1500–2000, rb, ab84892 Abcam; 1:500, rb, NB100-97823, Novusbio), ARL13B (1:200, ms, 73-287, Neuromab; rb, 17711-1-AP, Proteintech); TUBGCP2 (1:100, rb, 25856-1-AP, Proteintech), CEP70 (1:500, rb, ab227456, Abcam), acetylated tubulin (1:250, ms, ab24610, Abcam), CEP250 (1:200, rb, 14498-1-AP, Proteintech), Centrin-3 (1:500, rb, PA5-35865, Thermo), Centriolin (1:200, ms, sc-365521, Santa Cruz), RPGR (1:500, rb, HPA001593, Sigma Aldrich). Secondary Antibodies were Alexa488 goat anti-mouse (A11029, Molecular probes) and anti-rabbit (A11034, Molecular Probes), Alexa568 goat anti-mouse (A11031, Molecular probes) and anti-rabbit (A11036, Molecular Probes). For Western blot FLAGM2-HRP (Sigma) was used.

### Immunofluorescence Staining and Fluorescence Microscopy

150.000 cells/ml (hTERT-RPE1 single clones) were seeded onto an autoclaved glass slide in a 12-well plate. Cells were grown for 24 h and starved for 48 to 72 h to induce cilia assembly. For HEK293T cells, glass slides were coated using Poly-D-Lysine and starvation was done overnight prior fixation. Cells were fixed using 4% paraformaldehyde in PBS (PFA, 11,762.01000, Morphisto) for 10 min at RT and/or −20 °C methanol for 5 min and washed three times with PBS. 0.3% Triton containing PBS (PBST, Triton X-100, 1.08603.1000 Merck) was added for 5 min at RT, followed by blocking with 10% normal goat serum (NGS, S26–100 Ml, Merck) or 1% BSA (192,323,930, Roth) in PBS. Primary antibody incubation (1–2 at RT or 24 h overnight at 4 °C) was followed by PBS washing and secondary antibody incubation for 1 to 2 h at RT. Nuclei were stained with DAPI (62.248, Thermo Scientific). Zeiss Axio Imager Z1 ApoTome microscope (Carl Zeiss Microscopy GmbH, Germany) with an AxioCam MRm camera and 40× (NA 1.3) and 63 x (NA 1.4) oil immersion objectives lenses were used for imaging. Z-stacks were taken and further processed with Zeiss ZEN 2.6/3.0 Blue Edition (Carl Zeiss Microscopy GmbH, Germany).

### Ciliary Length and Ciliation Measurement

Three biological replicates with four technical replicates were incorporated into the analysis for ciliation and ciliary length. ARL13B for ciliary staining and DAPI for nuclei staining were used. For automated image analysis, the plugin ALPACA 1.0.1 (20) for Fiji (21) was applied. Analyses were done with unpaired Welch *t* test (GraphPad Prism 5 software) due to unequal variances. Three asterisks imply *p*-value <0.001. Error bars indicate mean with SEM.

### Viability/Proliferation Assay (Crystal Violet)

Triplicates of 7.000 cells/per well of 24-well plates were seeded on day zero in triplicates. Cells were fixed after 6, 24, 48, and 72 h. For each time point, 0.5 ml of 0.2% Crystal Violet (CV, Sigma Aldrich) in 20% Ethanol was used per well to fixate and stain the cells. Cells were washed three times and air dried at least ON. 1 ml 10% Acetic Acid/well was added plates were incubated at 200 rpm for 20 min at RT. The crystal violet absorption was measured using a Tecan Reader (Vilber) at a wavelength of 585 nm.

### Western Blot

Western blot was done as described before. Briefly, eluates were boiled at 95°C for 5 min, separated by 8% SDS-Page at 80V for 2-3h, and blotted to PVDF membranes (Millipore). After blocking with 5% milk/TBST solution, membranes were incubated with antibodies in 5% milk/TBST.

## Results

### Endogenously Tagged ALMS1 Localizes to the Centrosome and Basal Body

**Fig. 1:**
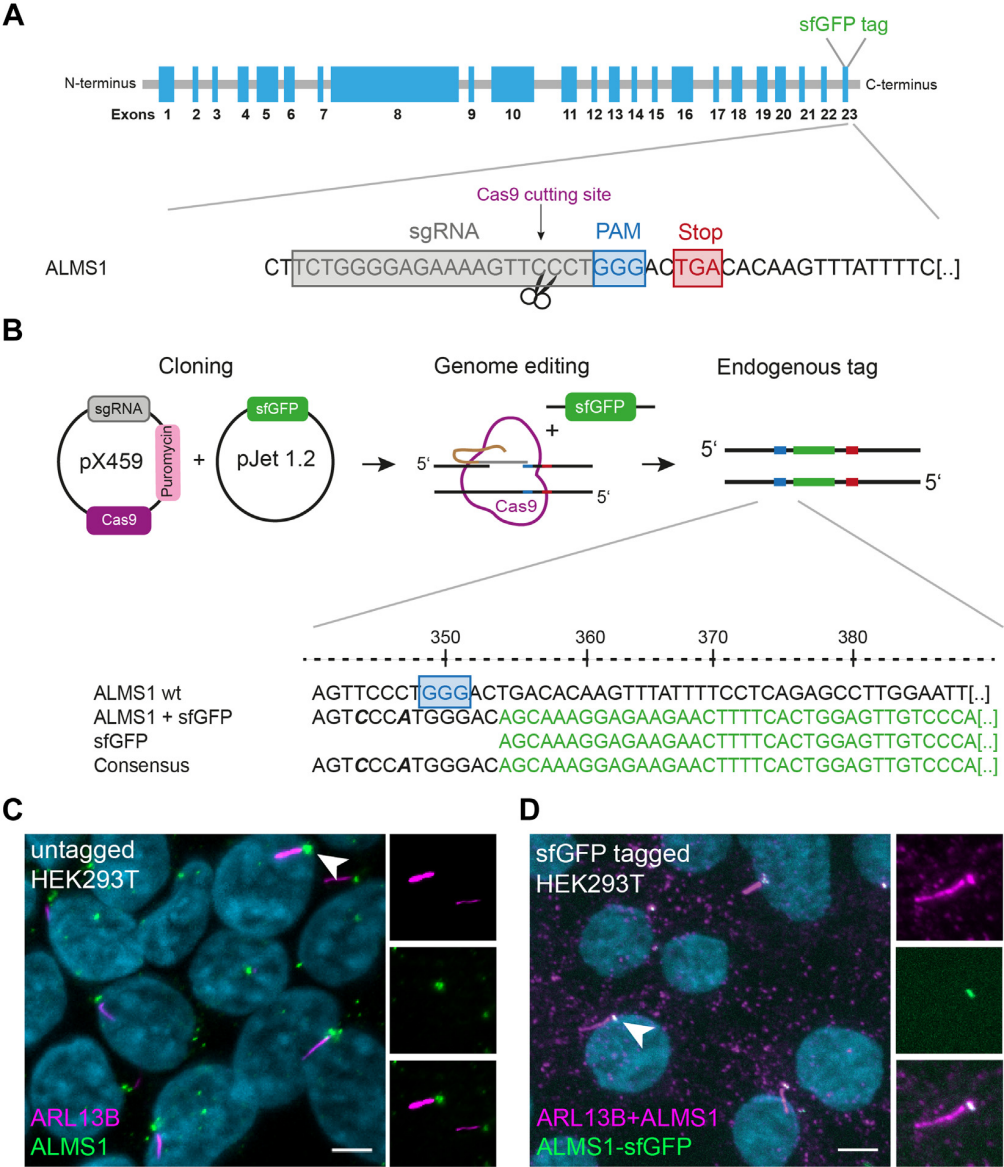
**Workflow of ALMS1 endogenous tagging in HEK293T cells using CRISPR/Cas9.***A*, schematic overview of the *ALMS1* gene with 23 exons (*blue*) and the site for tag insertion at the C-terminal end of *ALMS1* (*upper panel*). Enlarged the sgRNA targeting site (*grey*), PAM sequence (*blue*), and the stop codon (*red*) are depicted. Cas9 cuts (*purple*) three nucleotides upstream of the PAM sequence. *B*, simultaneous transfection of *ALMS1* targeting sgRNA cloned into the CRISPR/Cas9 (pX459.2) vector, and the sfGFP sequence harboring repair construct into HEK293T cells was performed. PCR-amplified products of ALMS1 CT region of HEK293T single clones were sent in for sequencing, confirming sfGFP tag insertion at the CT end of *ALMS1* (example shown in *lower panel*). *Bold italic* marked base pairs were wobbled to prevent repeated cutting after tag insertion. *C*, native ALMS1 localized at the basal body of cilia in HEK293T cells (G0 phase). ARL13B was used as a ciliary marker. ALMS1 is depicted in *green*, ARL13B in *magenta*, and DAPI in *light blue*. The scale bar measures 5 μm. *D*, endogenously sfGFP-tagged ALMS1 (ALMS1-sfGFP) in HEK293T single clones in the G0 phase. Cells were stained with ARL13B (*magenta*) as a ciliary marker and ALMS1 (*magenta*) as a basal body marker. DAPI is depicted in *light blue*. The scale bar measures 5 μm.

Despite the growing list of ALMS1 mutations, the molecular function is still elusive. Protein complex investigation provides a powerful tool to identify interaction partners of the protein of interest, which can be further validated functionally in a context-specific manner. However, ALMS1 (ENSG00000116127) represents a protein with ∼460 kDa of size, which hampered investigation of the full-length protein in the past (22, 23). Transient overexpression might lead to misfolded protein resulting in a long list of false-positive hits (24). Since the invention of the CRISPR/Cas9 technology, endogenous tagging of proteins already led to a deeper understanding of cilia protein function (25, 26, 27). Here, the CRISPR/Cas9 method was used to insert a superfolder (sf) GFP tag to the C-terminus (CT) of *ALMS1* in human embryonic kidney (HEK293T) cells to control localization to the ciliary BB and investigate protein interactions. The sfGFP tag was used due to its prediction to not influence the folding properties of the protein (28). A specific sgRNA targeting the nucleotide sequence before the stop codon of *ALMS1* was cloned (Fig. 1*A*). SgRNA was designed using the online tool CCTop (16) and chosen based on low off-target predictions in other exonic regions (supplemental Table S1). The sgRNA was cloned into the pSpCas9(BB)-2A-Puro vector (PX459, which was a gift from Feng Zhang) (29). For sfGFP tag insertion at the CT, a repair construct harboring homology arms of approximately 355 base pairs (bp) on each side of the sfGFP sequence was sub-cloned into the pJet plasmid. The homology arms contained wobbled base pairs (Fig. 1*B*, bold italic) in the sgRNA region to prevent repeated cutting after successful sfGFP sequence insertion. In addition, the homology arms were flanked by sgRNA-targeted sequences for linearization of the repair construct *in vivo* which is described to increase repair efficiency (30). After transfection, antibiotic, and single clone selection, a first screening for positive insertion of the tag in the *ALMS1* gene was performed using isolated genomic DNA for PCR (supplemental Fig. S1). Finally, the successful insertion of sfGFP tag was confirmed by Sanger sequencing (Fig. 1*B*). Out of 49 clones, seven clones showed homozygous C-terminal insertion of sfGFP (supplemental Fig. S1*C*).

ALMS1 is known to be localized to the centrosome in dividing and to the basal body (BB) of ciliated cells (4, 6, 31). To confirm the correct localization of endogenous ALMS1-sfGFP, three homozygous single clones and HEK293T untagged (wt) cells were co-stained for the ciliary marker ARL13B and ALMS1. Immunostaining revealed ALMS1-sfGFP localization at the BB of primary cilia in HEK293T cells comparable to wt cells, suggesting that tag insertion did not interfere with ALMS1 localization (Fig. 1, *C* and *D*, supplemental Figs. S4, and S5). In addition, basal body proteins like centriolin (CNTRL), centrosomal protein 250 kDa (CEP250), and γ-tubulin were co-localized to the GFP-positive ALMS1 signal at the base of the cilium (supplemental Figs. S4 and S5). Furthermore, ALMS1-sfGFP could be found in mitotic cells, exemplary at the spindle poles in metaphase, anaphase, and telophase (supplemental Fig. S3), as described before (4). Taken together, data indicate that ALMS1 was successfully tagged in HEK293T cells while maintaining ALMS1 and basal body marker localization, hence providing the ideal system to study ALMS1 interacting proteins.

### Complex Analysis Using Endogenously Tagged Cells Revealed New Interactions of ALMS1

**Fig. 2:**
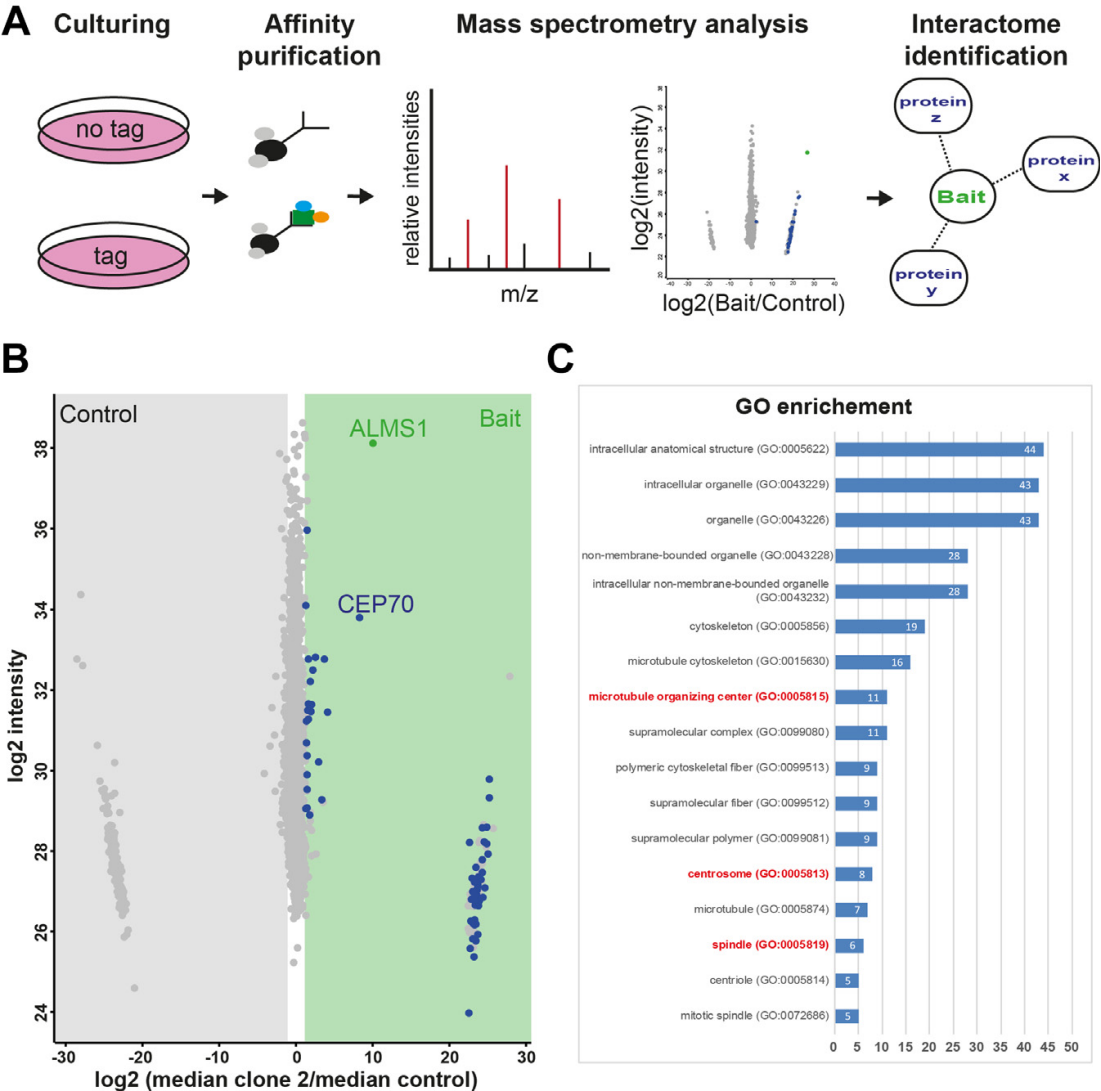
**Novel interaction partners of ALMS1 identified by mass spectrometry.***A*, ALMS1 tagged cells as well as control cells were used for GFP-based affinity purification, followed by mass spectrometry analysis to identify potential ALMS1 interaction partners. *B*, Scatter plot summarizing data for sfGFP-tagged ALMS1 clone 2. In total six biological replicates were analyzed for control and tagged HEK293T cells. Mass spectrometry data were analyzed using MaxQuant and Perseus. On the x-axis the log_2_ ratio of median ALMS1-sfGFP/median control and on the y-axis the log_2_ of their intensity is shown. Proteins that were more abundant in ALMS1 tagged samples are on the right with significantly enriched proteins marked in *blue* (Tier 2). *C*, GO enrichment analysis was performed using the knowledgebase provided by the Gene Ontology Consortium (http://geneontology.org/), here sorted by protein number present in the dataset and assigned to specific GO (more details in supplemental Table S5).

Three independent HEK293T single clones expressing endogenously sfGFP-tagged ALMS1 were used to investigate the protein-protein interaction network of ALMS1. HEK293T wildtype (wt) cells served as a control, for the determination of unspecific bound proteins. Here, six replicates of three ALMS1-sfGFP single clones and control cells were analyzed, respectively. Label-free quantification of mass spectrometry (MS) data was performed using MaxQuant (32), followed by a statistical evaluation using Perseus (17) (Fig. 2), as done before (25, 33). ALMS1 interactors were determined based on significance A FDR <0.05 (Benjamini-Hochberg outlier test) and a Student’s *t* test FDR <0.05 (Permutation-based, Tier 1 proteins). As Permutation-based FDR represents a very stringent statistical test, here proteins with a Student’s *t* test *p* < 0.05 are shown as well (Tier 2). Only proteins that passed both tests, significance A and Student’s *t* test, were considered to be specifically enriched in ALMS1 samples when compared to the control (Fig. 2*B* and supplemental Table S4). The bait ALMS1 was found with a high sequence coverage between 38% (clone 1) and 69% (clone 2) in ALMS1-sfGFP cells (supplemental Figs. S1 and S2). Proteins which were found as Tier 2 proteins in minimum two independent clones were defined as ALMS1 network candidates (supplemental Table S4, VENN diagram). In total, an overlap of 11 proteins was observed with all three clones, and 35 were significantly enriched in minimum two clones. For GO enrichment analysis the Gene Ontology knowledgebase provided by the Gene Ontology Consortium was used (34, 35). Enrichment analysis was done based on cellular component criteria, to highlight protein groups, which might be relevant for the centrosomal/BB-related function of ALMS1 (supplemental Fig. S2*C* and supplemental Table S5). In total, 16 are indicated as microtubule cytoskeleton proteins, nine are centrosome or MTOC associated proteins grouped together with ALMS1 (CCP110, PRKAR1A, CEP97, FSD1, CEP70, ASPM, HDAC6, NEURL4, AKAP11, HERC2).

**Fig. 3:**
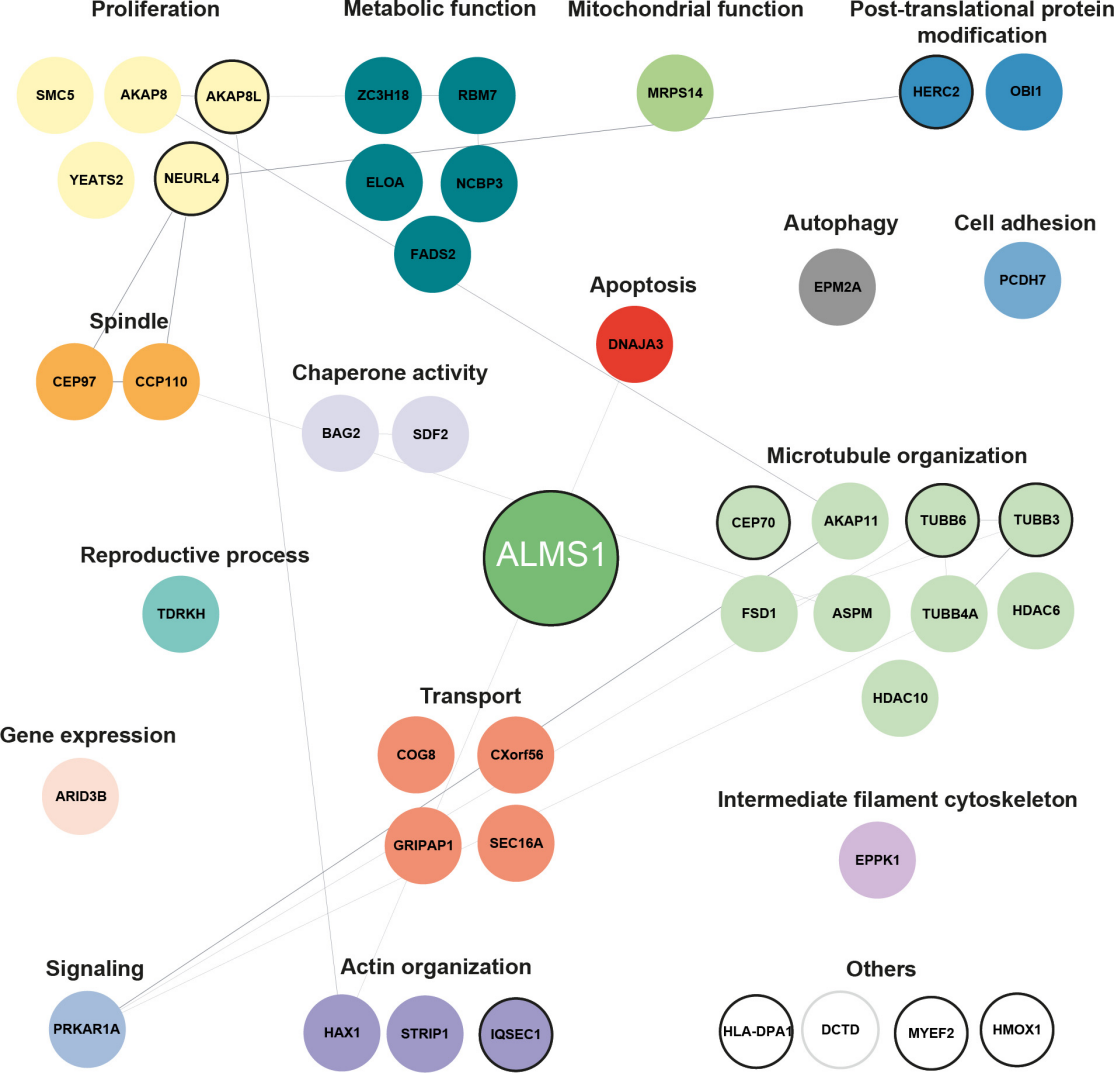
**ALMS1 interaction network.** A detailed network of interaction partners of ALMS1 linked to their function in cellular processes. Three independent ALMS1-sfGFP HEK293T cells were analyzed and compared to untagged control cells. Proteins were grouped in Tier 1 and Tier 2 based categories (Tier 1: Significance A (Benjamini-Hochberg) FDR <0.05) and permutation-based FDR <0.05; Tier 2: Significance A (Benjamini-Hochberg) FDR <0.05 and *p*-value <0.05). Proteins which were overlapping in minimum two ALMS1-sfGFP clone based on the Tier 2 category, were defined as ALMS1 complex proteins and included in the network. Proteins which were present as Tier 2 proteins with all three ALMS1-sfGFP clones are shown with bold circles. Network proteins were grouped according to their main function, as described on the UniProt Knowledgebase (the 2023_02 released version, (64)). Network was generated using experimental and functional interactions extracted from curated database using STRING and visualized in Cytoscape (18, 19).

In addition, based on the Uniprot Knowledge database (UniprotKB), Tier 2 proteins were grouped into functional clusters. Tier 2 proteins found are involved in metabolic function and proliferation, or participate in spindle, transport, and microtubule organization (Fig. 3).

For the first time, CEP70 has been identified as an interaction partner of ALMS1. CEP70 is known to be localized at the centrosome and BB of cilia and was highly significantly enriched in the protein complex analysis, pointing towards a strong interaction with ALMS1. CEP70 is described as influencing microtubule stability by regulation of tubulin acetylation *via* HDAC6 (36). Loss of CEP70 leads to left-right defects and reduced ciliation in zebrafish (37). Here, we wanted to validate ALMS1-CEP70 interaction and understand its role in ciliated cells in more detail using CRISPR/Cas9-mediated knockout cells.

### ALMS1-Deficient Cells Exhibit Shortened Cilia With Unaffected γ-Tubulin at the BB

**Fig. 4:**
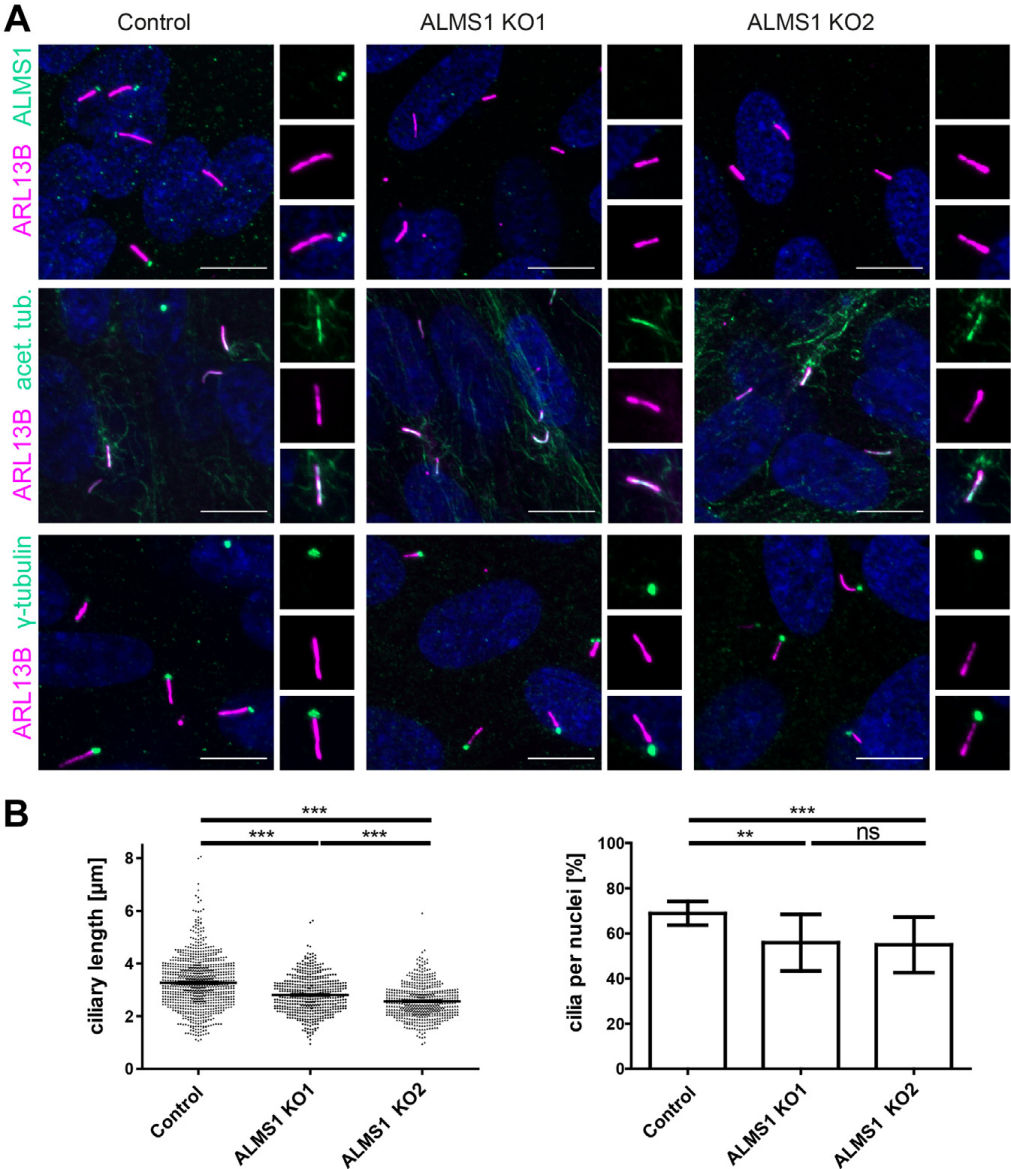
**ALMS1-deficient cells showed reduced ciliary length and number.***A*, ALMS1 KO1 (p.Glu2462Glyfs∗8, p.Ser2846Argfs∗10), ALMS1 KO2 (p.Asp2458Argfs∗7, p.Ser2846Lysfs∗38) and control hTERT-RPE1 cells were stained for ARL13B (*cilia, magenta*) and ALMS1, acetylated tubulin or γ-tubulin (*green*). In control cells, ALMS1 was detected at the basal body. In the two ALMS1 KO cell lines ALMS1 was lost at the cilium. No difference was observed for ARL13B, acetylated tubulin and γ-tubulin compared to control. DNA dye DAPI is marked in *blue*. Scale bar = 10 μm. *B*, ciliary length measurement with control and ALMS1 KO single clones. 905 cilia for control, 549 cilia for ALMS1 KO1 and 535 cilia for ALMS1 KO2 were measured. On the y-axis the ciliary length (μm) is shown. Three biological replicates (n = 3) with respectively four ApoTome pictures were used for analysis. An unpaired *t* test with Welch’s correction test was applied. Error bars indicate SEM. In addition, the ciliation rate was high significantly reduced in both ALMS1 KO cell lines compared to control.

To understand the functional relation of ALMS1 and its interactor CEP70, epistasis experiments with the downregulation of both proteins were considered. In the first step, ALMS1 KO cells were generated and phenotypically investigated. The CRISPR/Cas9 mediated loss of ALMS1 was done in human Telomerase Reverse Transcriptase-immortalized Retinal Pigmented Epithelial cells (hTERT-RPE1). HTERT-RPE1 cells were used due to their robust ciliation, cytoskeletal arrangement, and polarization (38). Specific sgRNAs targeting exons 8 and 10 of *ALMS1* (supplemental Tables S2 and S3) were used for transfection, followed by single clone selection and sequence verification by Sanger sequencing, as described for tag insertion before (supplemental Fig. S6). The ALMS1 KO led to frameshift mutations predicted to lead to an early stop (ALMS1 KO1: p.Asp2458Argfs∗7, p.Ser2846Lysfs∗38; ALMS1 KO2: p.Glu2462Glyfs∗8, p.Ser2846Argfs∗10). Two ALMS1 KO clones and control (treated with CRISPR/Cas9 without gene-specific sgRNA) were further phenotypically analyzed using Western blot and immunofluorescence microscopy. By Western blot, no ALMS1-specific band could be distinguished from the background (data not shown). In immunofluorescence staining, ALMS1 was detected at the base of the cilium in control cells, as expected. In ALMS1-deficient cells, ALMS1-specific staining was absent, which proved sufficient loss of ALMS1 at the cilium (Fig. 4*A*). Several ciliary and basal body markers were analyzed in these cells. No differences were seen for acetylated tubulin and ARL13B in the cilium, and for y-tubulin, Centrin-3, and PCM1 in control *versus* ALMS1 KO cells (Fig. 4*A* and supplemental Fig. S7), and as described before (12). The interactor CEP70 was not changed in localization at the centrosome, as well. However, BB staining of CEP250 was reduced upon loss of ALMS1 as previously described, which confirms the consistency of this specific phenotype (supplemental Fig. S7) (12).

Next, ciliary length and ciliation were investigated. Therefore, the ciliary marker ARL13B, which can be found at the ciliary membrane, was used for measurements (39) with ALPACA (Accumulation and Length Phenotype Automated Cilia Analysis) (20). In previous studies, it was shown, that the ciliary length varies enormously among ALMS1 knockdown (KD) or KO models and AS patient-derived fibroblasts compared to controls (4, 5, 11, 12, 13). In our study, ALMS1 deficient cells exhibit mild yet significantly shorter cilia compared to the control cells (ALMS1 KO1 vs control: t(1445) = 17.11, *p* < 0.0001, n = 3; ALMS1 KO2: t(1384) = 10.68, *p* < 0.0001, n = 3) (Fig. 4*B*). In total, 905 cilia were counted in control cells, 549 cilia for ALMS1 KO1 and 535 cilia in ALMS1 KO2 cells and analyzed using unpaired *t* test with Welch’s correction. The mean of ciliary length in ALMS1 KO1 cells was 2.562 μm ± 0.02653 and in ALMS1 KO2 was 2.805 μm ± 0.02982, while the mean of control cells was 3.272 μm ± 0.03188. In addition, the cilia number in relation to the number of nuclei was counted (ALMS1 KO1: t(20) = 3.927, *p* < 0.0008; ALMS1 KO2: t(18) = 3.489, *p* < 0.0026), resulting in a significant reduction of ciliation between ALMS1 KO2 (68,84% ± 1.581) and ALMS1 KO1 (54.91% ± 3.178) and control cells (68.84% ± 1.581). By comparing ciliation of ALMS1 KO1 (54,91 ± 3.178) and ALMS1 KO2 (55.88 ± 3.362), no difference was observed.

### Loss of CEP70 led to Reduced ALMS1 at the Basal Body in hTERT-RPE1 Cells

**Fig. 5:**
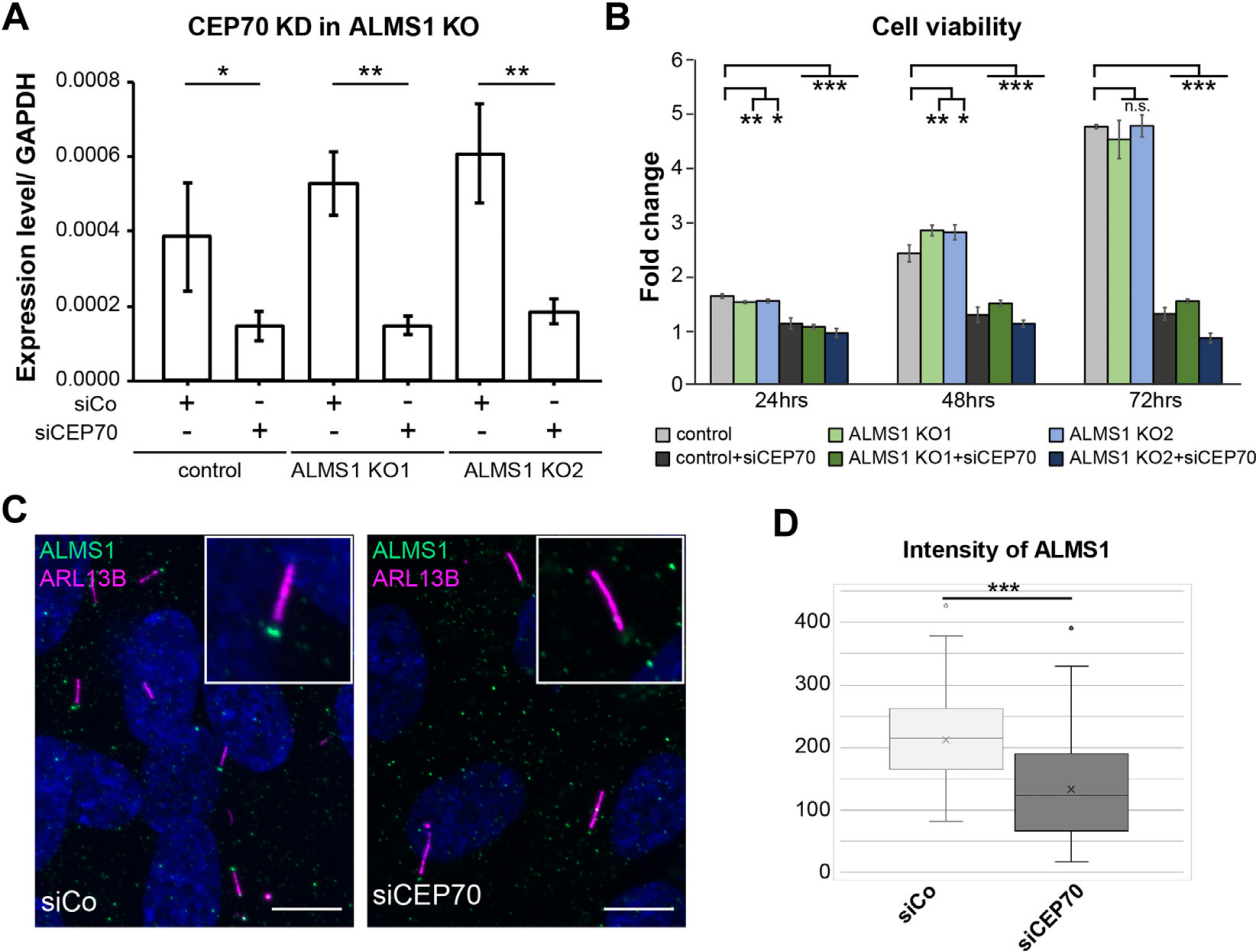
**CEP70 KD led to decreased ALMS1 at the BB.***A*, CEP70 KD was done in control cells and two ALMS1 KO cell lines. Four technical replicates for three biological replicates were measured, respectively. Transfection of 10 nM pooled siRNA targeting *CEP70* resulted in a significant reduction of *CEP70* mRNA. No difference was seen between the control and ALMS1 KO cells. *CEP70* qPCR primer spam from exon 9 to 11. *GAPDH* was used as a housekeeping gene for normalization. *B*, *Crystal violet* assay was performed using Cas-transfected control and ALMS1 KO hTERT-RPE1 cells. Three biological replicates and two technical replicates were measured after 24 h, 48 h, and 72 h, and fold change relative to the time point 0 (6 h after seeding) was determined. Significant differences compared to control cells based on the Student’s *t* test was calculated for each time point. CEP70 KD resulted in a very high significant reduction of cell viability. *C*, hTERT-RPE1 wt cells were transfected with control or CEP70 siRNA. After 48 h, cells were starved for 3 days and *cilia* and ALMS1 localization were investigated. ALMS1 is depicted in *green*, ARL13B is show in *magenta* and DNA dye DAPI in *blue*. Scale bar = 10 μm. *D*, the intensity of ALMS1 at the cilium was measured. A defined circular area for ALMS1 and five *circular areas* around the basal body for background subtraction were measured for each cilium. 89 cilia for control and 56 cilia for CEP70 siRNA transfected cells were measured. The ALMS1 intensity at the cilium was very high significantly reduced in CEP70 KD cells compared to control transfected cells. The significance was calculated based on the Student’s *t* test.

To investigate if CEP70 and ALMS1 interaction is relevant for overlapping processes, an epistasis experiment with the downregulation of both genes was envisaged. In a first attempt to generate double KO (dKO) cells lacking both CEP70 and ALMS1, no dKO clone could be verified and a high rate of cell loss was observed throughout the CRISPR/Cas9 mediated CEP70 KO approach (data not shown). For that reason, leveled knockdown (KD) of CEP70 was performed in control and ALMS1 KO cells. First, CEP70 siRNA-based KD was evaluated using different concentrations (5–50 nM, siCEP70) by qPCR (supplemental Fig. S8). No reduction of *CEP70* mRNA level was detected with 5 nM, whereas between 10 to 50 nM a gradual decrease of *CEP70* was observed. Next, it was investigated if ALMS1 KO cells show increased susceptibility to CEP70 KD when 10 nM siRNA targeting CEP70 was transfected. No difference in KD efficiency was observed 72 h after transfection (Fig. 5*A*).

To evaluate, if ALMS1 is influenced by CEP70 KD, ALMS1 was investigated in 10 nM siControl and 10 nM CEP70 siRNA transfected wt hTERT-RPE1 cells, respectively. Interestingly, CEP70 KD led to a very high significant reduction of ALMS1 intensity at the BB of cilia, indicating that CEP70 is involved in recruiting ALMS1 to or stabilization of ALMS1 at the BB (normalized intensity mean siCo = 212.9 ± 70.2 (89 cilia), normalized intensity mean siCEP70 = 132.9 ± 84.7 (56 cilia), *p* < 0.001; Fig. 5, *C* and *D*). As a general reduction of cells was observed in CEP70 KD experiments as well, cell viability was determined in wt and ALMS1 KO cells with or without CEP70 KD using the crystal violet assay, to investigate an epistatic effect upon concomitant loss of both BB proteins (Fig. 5*B*). A very high significant reduction in crystal violet staining in CEP70 KD cells compared to control transfected cells was seen at all three time points analyzed (24 h, 48 h, 72 h). No additional effect in the ALMS1 KO background was observed, hinting towards a CEP70 related phenotype independent of ALMS1, which has been described already by others (40, 41).

Conversely, in ciliary model organisms, CEP70 was shown to be dispensable for development and cell cycle regulation. In *Xenopus*, Cep70 regulates centriole amplification in multi-ciliated cells (42). In mice, it is involved in flagella formation and spermatogenesis (43). In zebrafish, Cep70 regulates left-right development and ciliogenesis (37). One could only speculate, that severe effects are prevented by redundancy during embryonic development.

To further investigate, which domain is involved in ALMS1 localization or stabilization at the ciliary basal body, and if overlapping complex proteins involved in cilia regulation can be found, we decided to analyze the CEP70 specific interactome in human cells (HEK293T), which might help to reveal candidate proteins being involved in ALMS1-dependent CEP70 function.

### CEP70 Interacts Domain Specific With ALMS1 and Other Centrosomal Proteins

Centrosomal localization of CEP70 is mainly conducted *via* its N-terminal (NT) half containing a coiled-coil domain, whereas expression of the TPR harboring CT is described to lead to no (41) or weak (42) centrosomal localization. Here, CEP70 full-length protein interactions were investigated, as well as the ALMS1-specific interacting domain should be defined. Despite the advantages of interactome investigation using endogenously tagged cells, as done for ALMS1, overexpression was performed for CEP70 analysis. A two-step genome editing procedure, endogenous tagging and large in-frame deletions, and two single clone selection procedures could result in clonal artifacts and would be difficult to control.

**Fig. 6:**
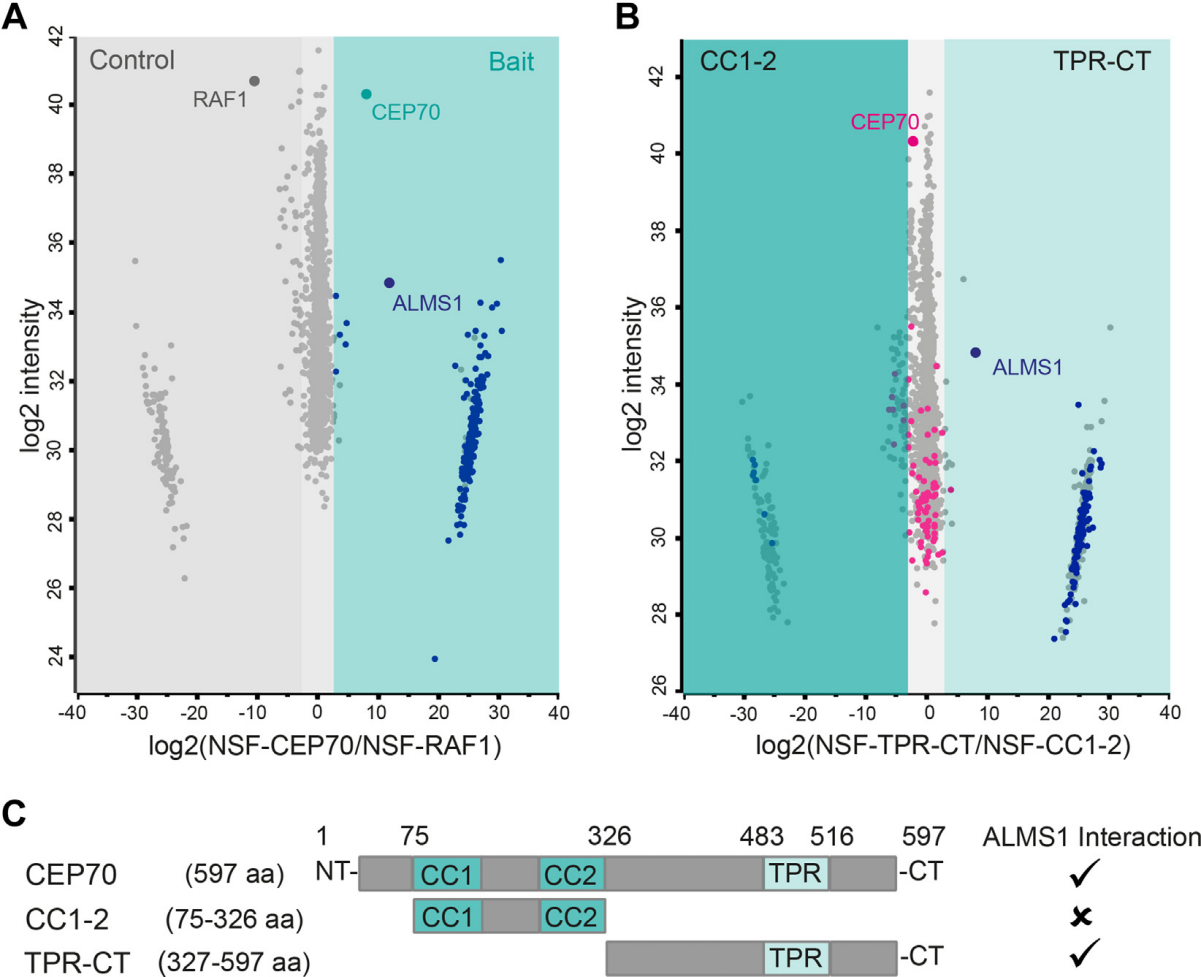
**CEP70 interacts with ALMS1 *via* its TPR domain.***A*, the scatter plot summarizes the results of six biological replicates of HEK293T cells transfected either with NSF-CEP70 (*cyan*) or the control (NSF-RAF1, *grey*). Mass spectrometry data were analyzed using MaxQuant and Perseus. On the x-axis log_2_ of the medians ratio and the y-axis log_2_ intensity are shown. The CEP70 protein (bait) is marked in *cyan*, control, and background in *gray* and significant CEP70 interactors (Tier 2) in *blue*. *B*, the scatter plot stresses the distribution of proteins, that are found with CC1-2 (*left side*) and/or with TPR-CT (*right side*) CEP70 constructs. Tier 2 proteins found with CEP70 full-length are marked in either *pink* (found in CC1-2 and TPR-CT samples with no significant difference) or *blue* (significant difference between CC1-2 and TPR-CT). *C*, schematic overview of full-length CEP70 (597 aa, CEP70) and specific CEP70 deletion constructs containing either the CC1 and CC2 (75–326 aa, CC1-2) domain or the TPR domain with the CT end (327–597 aa, TPR-CT). A positive identification of ALMS1 interaction with CEP70 is indicated with “✓”, whereas no interaction is shown with an “x”. CC = coiled coil, TPR = tetratricopeptide repeat, aa = amino acids, NT = N-terminus, CT = C-terminus, CEP70 = 597 aa (full-length), CC1-2 = CEP70 75 to 326 aa, TPR-CT = CEP70 327 to 597 aa.

**Fig. 7:**
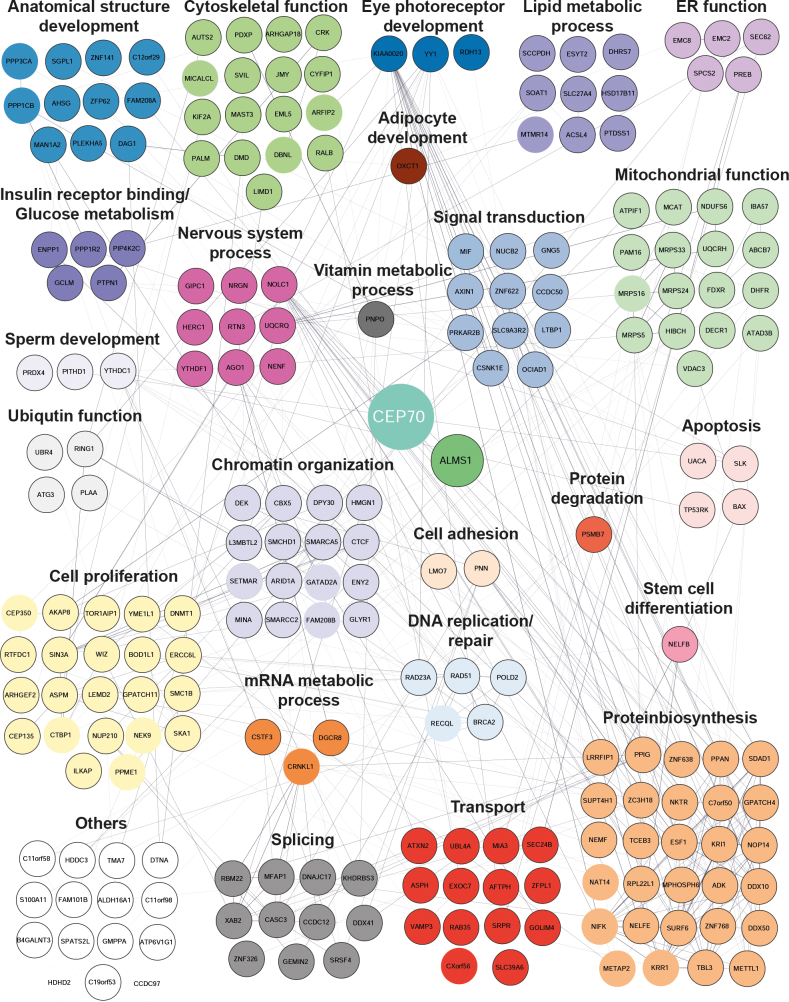
**Proteins interaction network of CEP70. CEP70 network proteins are involved in several cellular processes.** Proteins are grouped based on their main function (UniProt Knowledgebase, UniProtKB, 2023_02 released, (64)). Tier 1: Significant A *p* < 0.05 and permutation-based FDR *p* < 0.05 indicated with border painted in *black*; Tier 2: Significant A < 0.05 (Benjamini-Hochberg FDR <0.05) and *p*-value <0.05 indicated with no border painted. NACAP1 is predicted to be a pseudogene and is not included here. Network was generated using experimental and functional interactions extracted from curated database using STRING and visualized in Cytoscape (18, 19).

CEP70 full-length protein (CEP70, ENST00000264982.8, CEP70-201, CCDS3102) has 597 amino acids (aa). CEP70 fragments containing either the two coiled-coil domains (CC1-2, 75-326 aa) with 251 aa or the TPR domain and the CT end (TPR-CT, 327-597 aa) (Fig. 6*C*) were cloned into a N-terminal Strep/FLAG (NSF) plasmid, as described before (44). Transfection of CEP70 fragments as well as CEP70 full-length was followed by affinity-based protein complex purification and data-dependent analysis. In total, six replicates per condition (cilia-independent control (RAF1), CEP70) were analyzed. Student’s *t* test and significance A were applied to identify significantly enriched proteins, as described before (25) (Fig. 6*A*). Here, novel interaction partners of CEP70 were defined, which were subdivided into Tier 1 (Permutation based FDR *p* < 0.05) and Tier 2 (*p*-value <0.05) (Fig. 7). 202 proteins were found for Tier 1 and additionally 23 proteins referred to Tier 2. Using the Gene Ontology Resource as done before, did not give any enrichment of microtubule or centrosome-specific clusters (supplemental Table S7). When using UniProtKB for function-related clustering, 29 proteins were found with a cilia-related role (supplemental Fig. S9). Interestingly, CEP135 was found to be a CEP70 interactor, which is already listed in the BioGRID database, and represents a centrosomal protein regulating CEP250 and centriolar satellite localizations (45, 46, 47). In addition, CEP350 was enriched as a Tier 2 protein, which is described to serve as a scaffold protein involved in centriole function and early ciliogenesis (48, 49, 50). Other significantly enriched interaction partners are involved in transport, mitosis, signaling processes, mitochondrial function and show eye-related, nervous system and glucose metabolism functions (Fig. 7 and supplemental Table S6). Interestingly, ALMS1 was found with a sequence coverage of 28.3% (Tier 1) as an interactor of CEP70, underlining the strong interaction between the two proteins (supplemental Fig. S10).

Next, CEP70-specific interactors were further validated using the data obtained by affinity purification of deletion constructs. Domain-specific interactions were determined when comparing CEP70 full-length and TPR-CT or CC1-2, respectively. Both-sided statistical test was applied in this case, and only CEP70 interactors, which were already defined before (Tier 1, CEP70 full-length against control), were considered. In total 122 proteins were reduced in CC1-2 samples, and seven proteins showed significant decreased binding in TPR-CT samples (supplemental Table S6). When analyzing the CEP70 fragments, ALMS1 was identified as a significant interactor in TPR-CT but not in CC1-2 hinting towards an ALMS1-independent localization of CEP70 to the BB (supplemental Fig. S6*B*). In total, 18 ciliary proteins were decreased in binding in CC1-2 samples, including the centrosomal protein CEP135. Four CEP70 interactors are involved in apoptosis regulation and four in ubiquitin function, respectively. Six proteins were significantly changed in binding in TPR-CT parallel with ALMS1: UBR4, ATG3, PLAA, UACA, TP53PK, and BAX, which might be involved in CEP70-dependent cell death leading to reduced cell number observed in CEP70 KD as well as in epistasis experiments described before.

Taken together, the data gained in this study give insight into CEP70 and ALMS1 interaction patterns. New ciliary clusters could be described which might be involved in CEP70-dependent ALMS1 localization and ciliary function.

## Discussion

Disease-related mutations or loss of ALMS1 in different model organisms led to phenotypes that highly vary in symptom spectrum and severity (4, 10). Despite the involvement of ALMS1 in different cellular processes, the mechanism at the centrosome leading to ciliary defects remains elusive (4, 22, 51, 52, 53, 54). To get insights into functional relevant protein interactions, the ALMS1 protein complex was investigated here using endogenously tagged cells. An N-terminal tag design and insertion was not feasible due to a GC rich region and repetitive sequences of ALMS1, why the sfGFP tag was inserted C-terminally (CT). Another benefit of introducing a CT tag is that many transcript variants of ALMS1 were covered (55). Rather than transfecting overexpression constructs, endogenous tagging is closer to the physiological protein level and is predicted to not alter protein interactions (24). Furthermore, benefits of endogenously tagged proteins for interactome studies are the ability to investigate and analyze protein function in full-length and in a native context (56). In addition, no antibodies are needed, could show a low affinity to the beads or unspecific binding (57). Making use of sfGFP fluorescence, we could confirm correct centrosomal ALMS1 localization (31) and provide an ALMS1-specific interactome (Fig. 1, Fig. 2, Fig. 3, supplemental Fig. S5, and supplemental Table S4).

Go enrichment analysis indicated cellular component clusters at the centrosome and the MTOC (supplemental Table S5), with NEURL4, CEP70, and HERC2 stably found in all three tagged single clones. Recently, ALMS1 and exactly these three proteins were found to be centrosomal proteins in different cell types (58). CEP70, described by Shi *et al* in 2011, is involved in microtubule organization and stabilization by regulating tubulin acetylation *via* the histone deacetylase 6 (HDAC6) (31, 36). However, HDAC6 and 10 were significantly enriched in ALMS1 samples, but not found in CEP70 interaction data in the study presented here. It can only be speculated, that ALMS1 might be involved in CEP70-dependent regulation of tubulin acetylation. Furthermore, CEP70 interacts with its coiled-coil domains with γ-tubulin, which in turn assures a proper localization to the centrosome (41). CEP70 has to be de-ubiquitinylated to enable stable ciliogenesis (59). The ubiquitin carboxyl-terminal hydrolase CYLD has been described to be involved in this process, however, we did not find this protein in any of our datasets hinting towards a transient interaction. Interestingly, E3 ubiquitin-protein ligase HERC2 was enriched in the ALMS1 interactome. HERC2 is shown to be involved in ciliogenesis by ubiquitinylation and degradation of CCP110, which is part of our ALMS1 network as well (60). Recently it was shown, that NEURL4 which is a stable interactor found with all three ALMS1-sfGFP clones, is necessary for CP110 degradation and therewith cilia formation, as well (61). These findings indicate, that ALMS1 is involved in the transport or localization of NEURL4 and HERC2, which regulate CCP110 degradation and stabilize cilia formation. However, cilia are not lost but cilia length is mildly reduced in ALMS1-deficient cells, leading to the assumption, that ALMS1 might be involved but not essential for cilia assembly, which could be regulated by redundant processes.

The ciliary length in ALMS1 mutant models is highly discussed and either suggests a direct or an indirect role of ALMS1 in cilia assembly (4, 11, 12, 13, 15, 22). In 2021, Álvarez-Satta *et al* could show longer and bent cilia in their ALMS1 knockdown model in hTERT-RPE1 cells (51), while ALMS1 deficient cells investigated here exhibit shorter cilia. Differences in experimental setup could lead to disparities in the observed phenotype. Álvarez-Satta *et al* used inter alia an ALMS1 KD model in hTERT-RPE1 cells (51). It is up to speculation, if genotype-phenotype correlations add variation to ciliary length control, as the protein level of ALMS1 in an isoform-specific manner remains difficult. Hence, different mutations may cause a variety of phenotypical features and therefore influence the related disease AS. Mutations with a severe effect on protein structure or function might lead to more grave organ phenotypes.

Knockdown of CEP70 led to a severe reduction of the cell number (Fig. 5), as shown before (40, 41), which seemed to be an ALMS1 independent effect. However, CEP70 protein complex analysis revealed ciliary proteins as well as apoptosis-regulating proteins, which could be candidate proteins being involved in cell loss upon CEP70 KD.

Interestingly, ALMS1 and other ciliary proteins were lost when a truncated CEP70 fragment lacking the CC domains was used (Fig. 6*B*). As the two CC domains of CEP70 are essential for proper centrosomal localization of CEP70 (41, 42) these results indicate an ALMS1-independent localization of CEP70 to the centrosome, which we could confirm based on unaffected CEP70 localization in ALMS1 KO cells. Furthermore, CEP70 KD in hTERT-RPE1 wt cells showed a reduced or no ALMS1 signal at the BB of cilia, which suggests a regulating or stabilizing function of CEP70 on ALMS1 (Fig. 5).

Comparison between ALMS1 and CEP70 interaction data revealed five proteins that were found in both network datasets (AKAP8, ASPM, CXorf56, TCEB3 and ZC3H18) making them interesting candidates for overlapping CE70-ALMS1 complex function.

Other interesting CEP70 interactors are involved for example in insulin receptor binding, which could also serve as a link to the ALMS phenotypic occurrence of Type 2 diabetes. Ectonucleotide pyrophosphatase/phosphodiesterase family member 1 (ENPP1) was associated with insulin resistance by inhibiting the activity of insulin receptor (IR) and concomitantly preventing downstream signaling (62).

## Data Availability

The mass spectrometry proteomics data have been deposited to the ProteomeXchange Consortium *via* the PRIDE partner repository with the dataset identifier PXD046401 (63).

## Supplemental data

This article contains supplemental data.

## Conflict of interest

The authors declare no conflict of interest. The funders were not involved in the analyses or interpretation of data; in the writing of the manuscript or in the decision to publish the results.
