# Supplementary material for: Interactome Analysis Reveals a Link of the Novel ALMS1-CEP70 Complex to Centrosomal Clusters
Source: Mol Cell Proteomics. 2023 Dec 18;23(1):100701. doi: 10.1016/j.mcpro.2023.100701 (PMC10820798; doi:10.1016/j.mcpro.2023.100701)
Supplement: Supplement material [file mmc1.docx]

**Interactome analysis reveals a link of the novel ALMS1-CEP70 complex to centrosomal clusters**

Franziska Woerz^1^*, Felix Hoffmann^1^, Shibu Antony^1^, Sylvia Bolz^1^, Mohamed Ali Jarboui^1^, Katrin Junger^1^, Franziska Klose^1^, Isabel F. Stehle^1^, Karsten Boldt^1^, Marius Ueffing^1^ and Tina Beyer^1^*

^1^ Eberhard Karls University Tübingen, Institute for Ophthalmic Research, Elfriede-Aulhorn-Str. 7, University of Tübingen, 72076 Tübingen, Germany

* Correspondence: franziska.woerz@uni-tuebingen.de, F.W.; tina.beyer@uni-tuebingen.de, T.B.

**Supplemental Material**

Table S1 Predicted off-target effects of sgRNA ALMS1 exon 23

SgRNAs were designed using CCTop and were chosen with low off-target prediction. In this table 20 off-targets, with the highest risk, are depicted. Exons (E) are marked in red, Introns (I) in yellow and in (-) green.

| **Coordinates** | **strand** | **MM** | **target_seq** | **PAM** | **distance** | | **gene name** | **gene id** |
| --- | --- | --- | --- | --- | --- | --- | --- | --- |
| [chr2:73609585-73609607](http://genome.ucsc.edu/cgi-bin/hgTracks?db=hg38&position=chr2:73609585-73609607) | + | 0 | TCTGGGGA[GAAAAGTTCCCT] | GGG | 0 | E | ALMS1 | [ENSG00000116127](http://www.ensembl.org/Gene/Summary?g=ENSG00000116127) |
| [chr7:39506451-39506473](http://genome.ucsc.edu/cgi-bin/hgTracks?db=hg38&position=chr7:39506451-39506473) | + | 4 | **ATCT**GGGA[GAAAAGTTCCCT] | AGG | 13356 | - | POU6F2 | [ENSG00000106536](http://www.ensembl.org/Gene/Summary?g=ENSG00000106536) |
| [chr6:67688885-67688907](http://genome.ucsc.edu/cgi-bin/hgTracks?db=hg38&position=chr6:67688885-67688907) | + | 4 | T**AGT**GG**T**A[GAAAAGTTCCCT] | GGG | 63421 | - | Y_RNA | [ENSG00000206672](http://www.ensembl.org/Gene/Summary?g=ENSG00000206672) |
| [chr18:53753297-53753319](http://genome.ucsc.edu/cgi-bin/hgTracks?db=hg38&position=chr18:53753297-53753319) | - | 3 | **A**CTGG**A**GA[G**G**AAAGTTCCCT] | AGG | NA | - | NA | NA |
| [chr11:5199273-5199295](http://genome.ucsc.edu/cgi-bin/hgTracks?db=hg38&position=chr11:5199273-5199295) | + | 4 | TC**A**G**TTT**A[GAAAAGTTCCCT] | TGG | 440 | - | OR51V1 | [ENSG00000176742](http://www.ensembl.org/Gene/Summary?g=ENSG00000176742) |
| [chr6:39240540-39240562](http://genome.ucsc.edu/cgi-bin/hgTracks?db=hg38&position=chr6:39240540-39240562) | - | 4 | **CT**TG**A**GGA[G**T**AAAGTTCCCT] | GGG | 11090 | - | KCNK5 | [ENSG00000164626](http://www.ensembl.org/Gene/Summary?g=ENSG00000164626) |
| [chr14:65997912-65997934](http://genome.ucsc.edu/cgi-bin/hgTracks?db=hg38&position=chr14:65997912-65997934) | - | 4 | **AT**TGGG**A**A[**C**AAAAGTTCCCT] | GGG | 6521 | I | CTD-2014B16.3 | [ENSG00000258847](http://www.ensembl.org/Gene/Summary?g=ENSG00000258847) |
| [chr10:123892280-123892302](http://genome.ucsc.edu/cgi-bin/hgTracks?db=hg38&position=chr10:123892280-123892302) | + | 4 | T**TCT**GGGA[G**T**AAAGTTCCCT] | GGG | 296 | I | CPXM2 | [ENSG00000121898](http://www.ensembl.org/Gene/Summary?g=ENSG00000121898) |
| [chr10:75893633-75893655](http://genome.ucsc.edu/cgi-bin/hgTracks?db=hg38&position=chr10:75893633-75893655) | + | 4 | T**G**TG**T**G**TT**[GAAAAGTTCCCT] | GGG | 10108 | I | C10orf11 | [ENSG00000148655](http://www.ensembl.org/Gene/Summary?g=ENSG00000148655) |
| [chrX:52978988-52979010](http://genome.ucsc.edu/cgi-bin/hgTracks?db=hg38&position=chrX:52978988-52979010) | - | 4 | **G**CTGG**CC**A[G**C**AAAGTTCCCT] | TGG | 13007 | I | FAM156A | [ENSG00000268350](http://www.ensembl.org/Gene/Summary?g=ENSG00000268350) |
| [chr17:41327061-41327083](http://genome.ucsc.edu/cgi-bin/hgTracks?db=hg38&position=chr17:41327061-41327083) | - | 4 | TC**A**GG**CC**A[G**G**AAAGTTCCCT] | GGG | 24 | I | TBC1D3P7 | [ENSG00000233014](http://www.ensembl.org/Gene/Summary?g=ENSG00000233014) |
| [chr10:130485965-130485987](http://genome.ucsc.edu/cgi-bin/hgTracks?db=hg38&position=chr10:130485965-130485987) | - | 3 | T**G**TGG**A**GA[GAAA**G**GTTCCCT] | AGG | 2811 | - | RP11-540N6.1 | [ENSG00000236303](http://www.ensembl.org/Gene/Summary?g=ENSG00000236303) |
| [chr22:35354089-35354111](http://genome.ucsc.edu/cgi-bin/hgTracks?db=hg38&position=chr22:35354089-35354111) | + | 1 | TCTGGGGA[GAAAAG**G**TCCCT] | GGG | 6095 | - | TOM1 | [ENSG00000100284](http://www.ensembl.org/Gene/Summary?g=ENSG00000100284) |
| [chr5:128783191-128783213](http://genome.ucsc.edu/cgi-bin/hgTracks?db=hg38&position=chr5:128783191-128783213) | + | 3 | **AT**TGGGGA[GAAAA**C**TTCCCT] | TGG | 40362 | I | CTC-573M9.1 | [ENSG00000248634](http://www.ensembl.org/Gene/Summary?g=ENSG00000248634) |
| [chr8:124380736-124380758](http://genome.ucsc.edu/cgi-bin/hgTracks?db=hg38&position=chr8:124380736-124380758) | - | 4 | TC**AA**GG**A**A[GAA**C**AGTTCCCT] | TGG | 8044 | - | TMEM65 | [ENSG00000164983](http://www.ensembl.org/Gene/Summary?g=ENSG00000164983) |
| [chr2:149788325-149788347](http://genome.ucsc.edu/cgi-bin/hgTracks?db=hg38&position=chr2:149788325-149788347) | - | 4 | **AA**TGGGGA[GA**TG**AGTTCCCT] | GGG | 15835 | I | AC007364.1 | [ENSG00000162947](http://www.ensembl.org/Gene/Summary?g=ENSG00000162947) |
| [chr8:41674694-41674716](http://genome.ucsc.edu/cgi-bin/hgTracks?db=hg38&position=chr8:41674694-41674716) | - | 4 | **G**C**A**GGG**A**A[GAAAA**C**TTCCCT] | GGG | 1782 | I | ANK1 | [ENSG00000029534](http://www.ensembl.org/Gene/Summary?g=ENSG00000029534) |
| [chr11:117745399-117745421](http://genome.ucsc.edu/cgi-bin/hgTracks?db=hg38&position=chr11:117745399-117745421) | - | 4 | **CAA**GGGGA[GAAAAG**C**TCCCT] | TGG | 31370 | I | DSCAML1 | [ENSG00000177103](http://www.ensembl.org/Gene/Summary?g=ENSG00000177103) |
| [chr1:119175437-119175459](http://genome.ucsc.edu/cgi-bin/hgTracks?db=hg38&position=chr1:119175437-119175459) | + | 4 | TC**A**G**A**GGA[**C**AAA**G**GTTCCCT] | GGG | 5931 | I | RP11-418J17.1 | [ENSG00000231365](http://www.ensembl.org/Gene/Summary?g=ENSG00000231365) |
| [chrX:132084802-132084824](http://genome.ucsc.edu/cgi-bin/hgTracks?db=hg38&position=chrX:132084802-132084824) | - | 4 | **G**CTGG**CC**A[GAAAA**C**TTCCCT] | AGG | 217 | I | FRMD7 | [ENSG00000165694](http://www.ensembl.org/Gene/Summary?g=ENSG00000165694) |


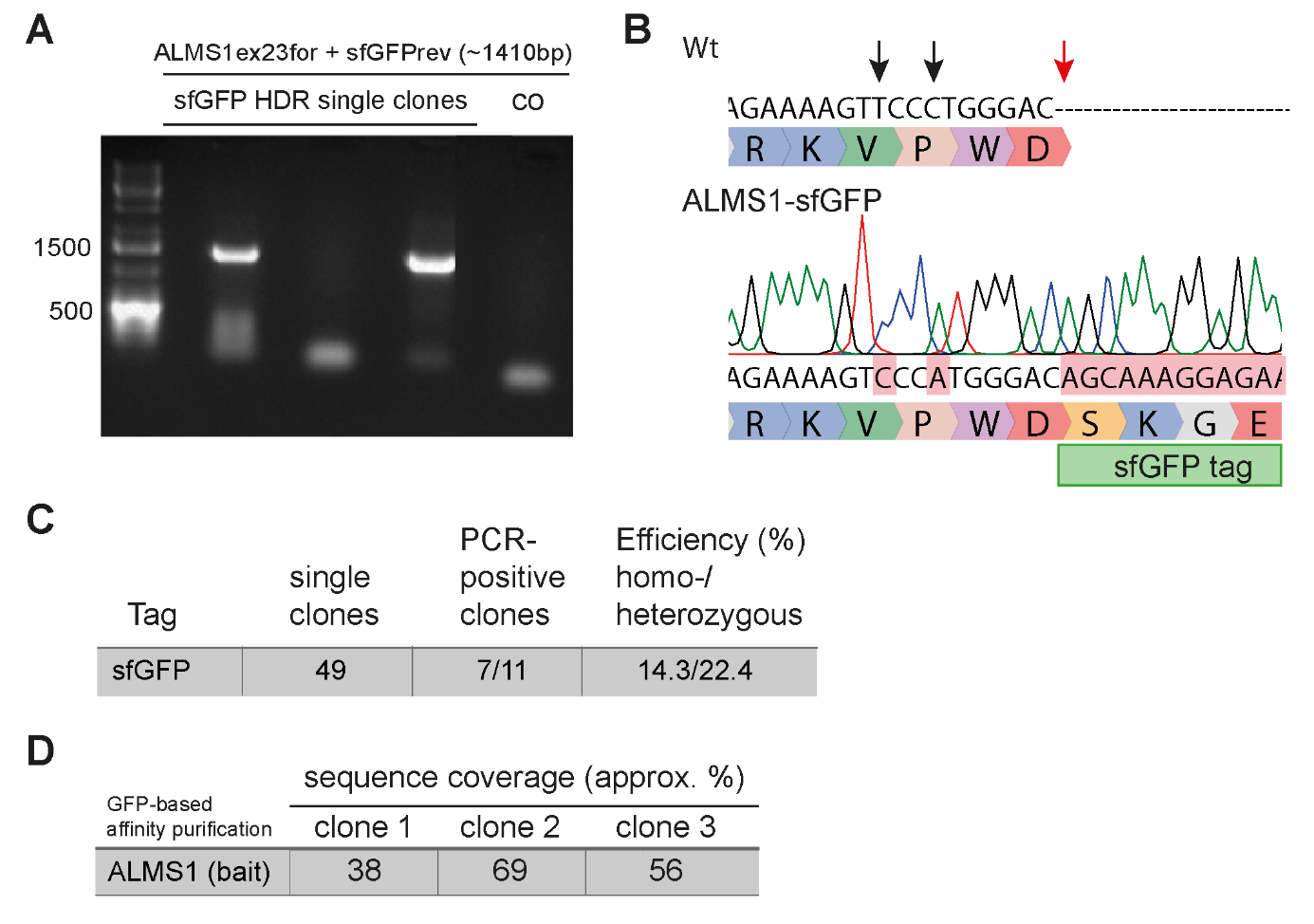
Figure S1 Verification of endogenously tagged ALMS1

(A) PCR and a subsequent agarose gel electrophoreses were conducted with CRISPR/Cas9-treated HEK293T sfGFP knock-in single clones. A forward primer for exon 23 and a reverse primer for sfGFP were used for all samples. SfGFP positive single clones show prominent bands at a height of approximately 1410 bp. No corresponding band was seen in Cas9 (without gene specific sgRNA) transfected control single clones.

(B) Nucleotide and amino acid sequence of ALMS1 wt (top panel) and ALMS1-sfGFP (bottom panel) are depicted. Black arrows indicate wobbled nucleotides, red arrow indicates the insertion site of the sfGFP sequence into the ALMS1 gene. Wt sequence was provided by ensemble.org (ENST00000613296.6, ALMS1-205, CCDS42697).

(C) The summary table with the number of single clones in total and positively tagged single clones is given. In total 49 single clones were picked, while seven homozygous and 11 heterozygous ALMS1-sfGFP single clones

were identified with an efficiency of 14.3 to 22.4%, respectively.

(D) ALMS1-sfGFP was purified performing GFP-affinity purification for three independent single clones. Protein digest was followed by mass spectrometry measurement. Mean sequence coverage out of six biological replicates was calculated for each clone.

**
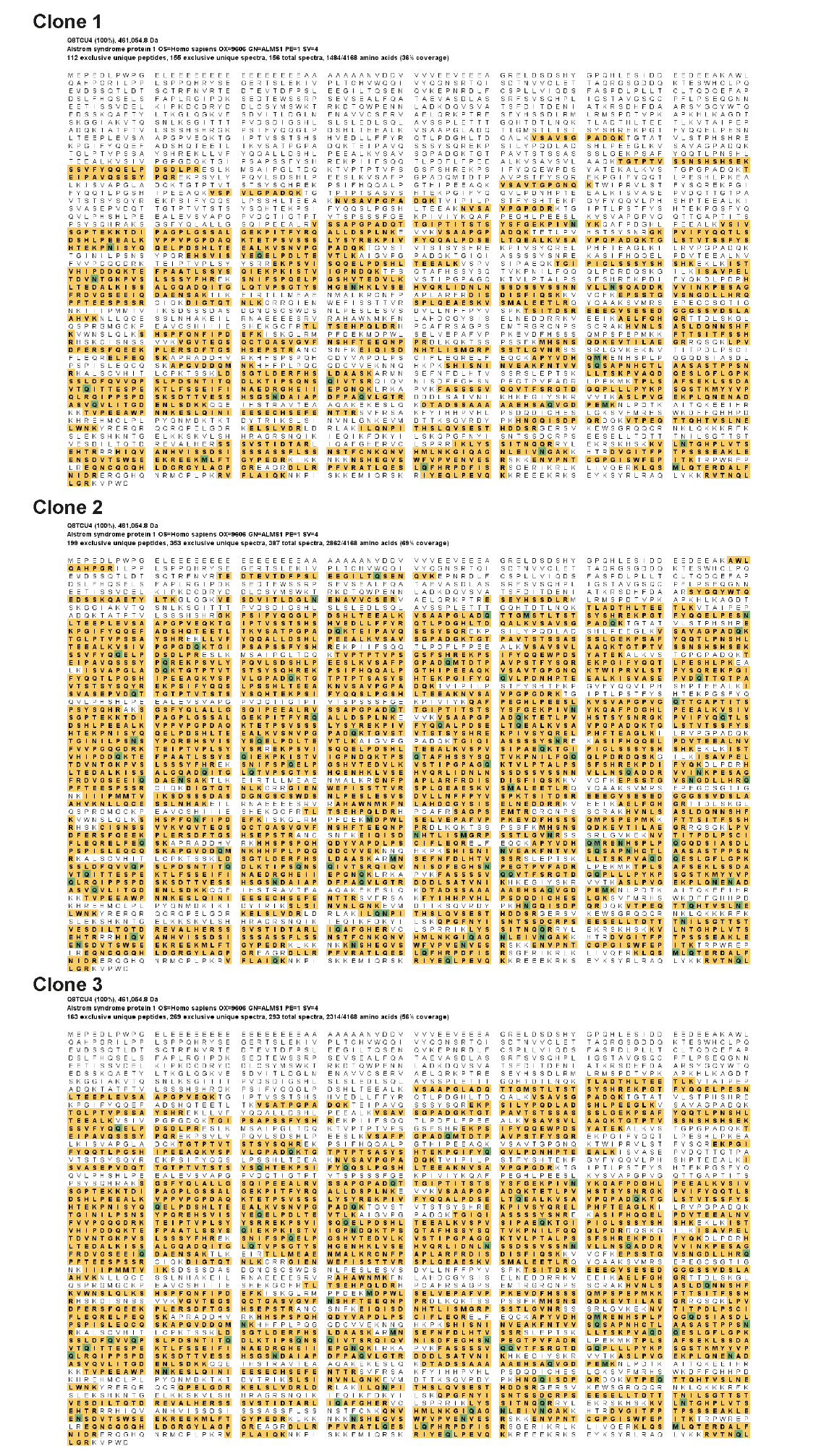
**

**Figure S2 ALMS1-sfGFP protein coverage**

Localization of identified peptides (exclusive unique peptides) is shown. Amino acids matched to MS/MS spectrum are marked in yellow. Amino acids with a post-translational modification are shown in green.


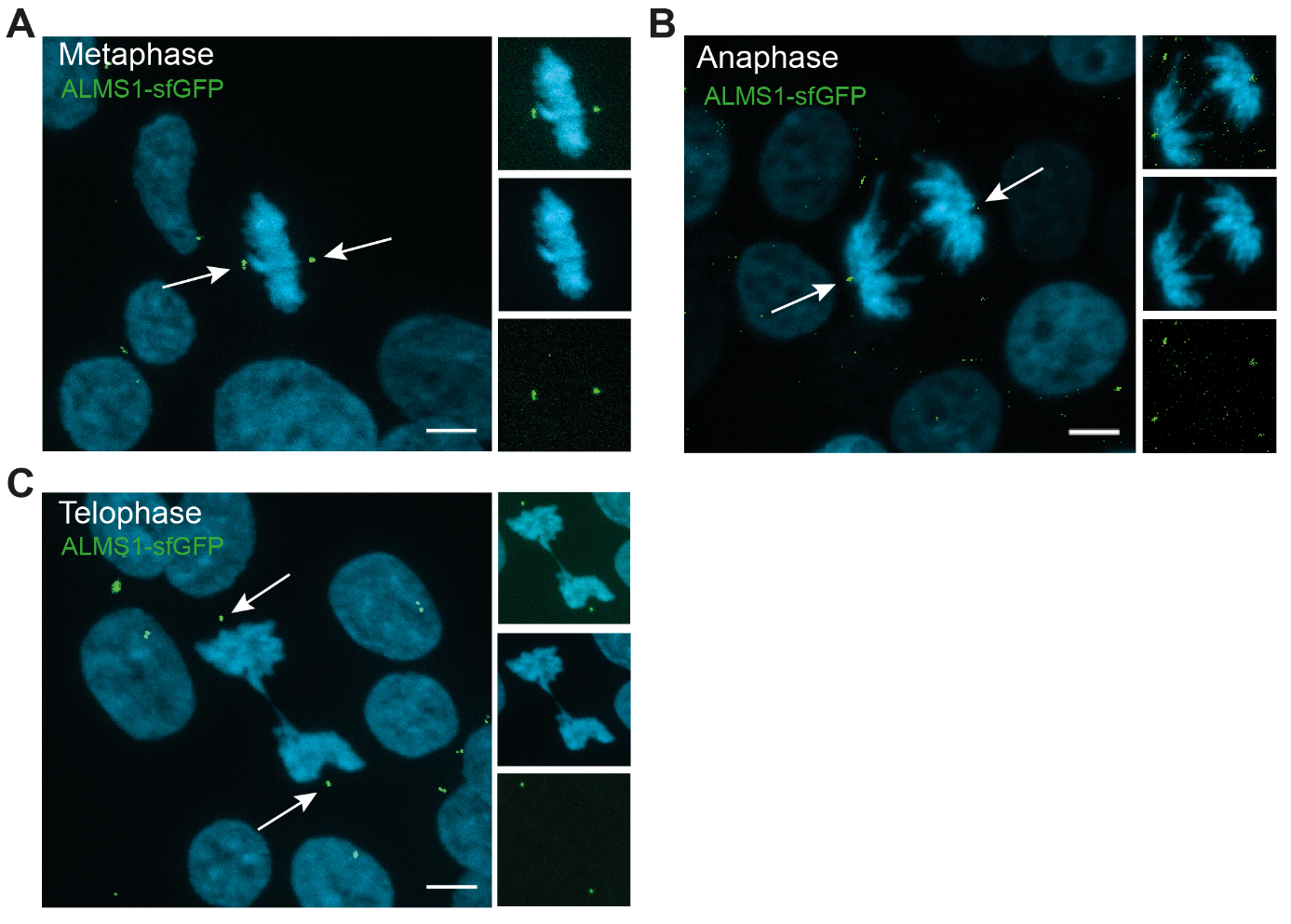


Figure S3 ALMS1-sfGFP localization in mitotic HEK293T cells

Endogenously sfGFP tagged ALMS1 (ALMS1-sfGFP) in different mitosis phases (Metaphase (A), Anaphase (B) and Telophase (C)) is shown. The arrows indicate ALMS1-sfGFP. DAPI is depicted in light blue. The scale bar measures 5µm.

**
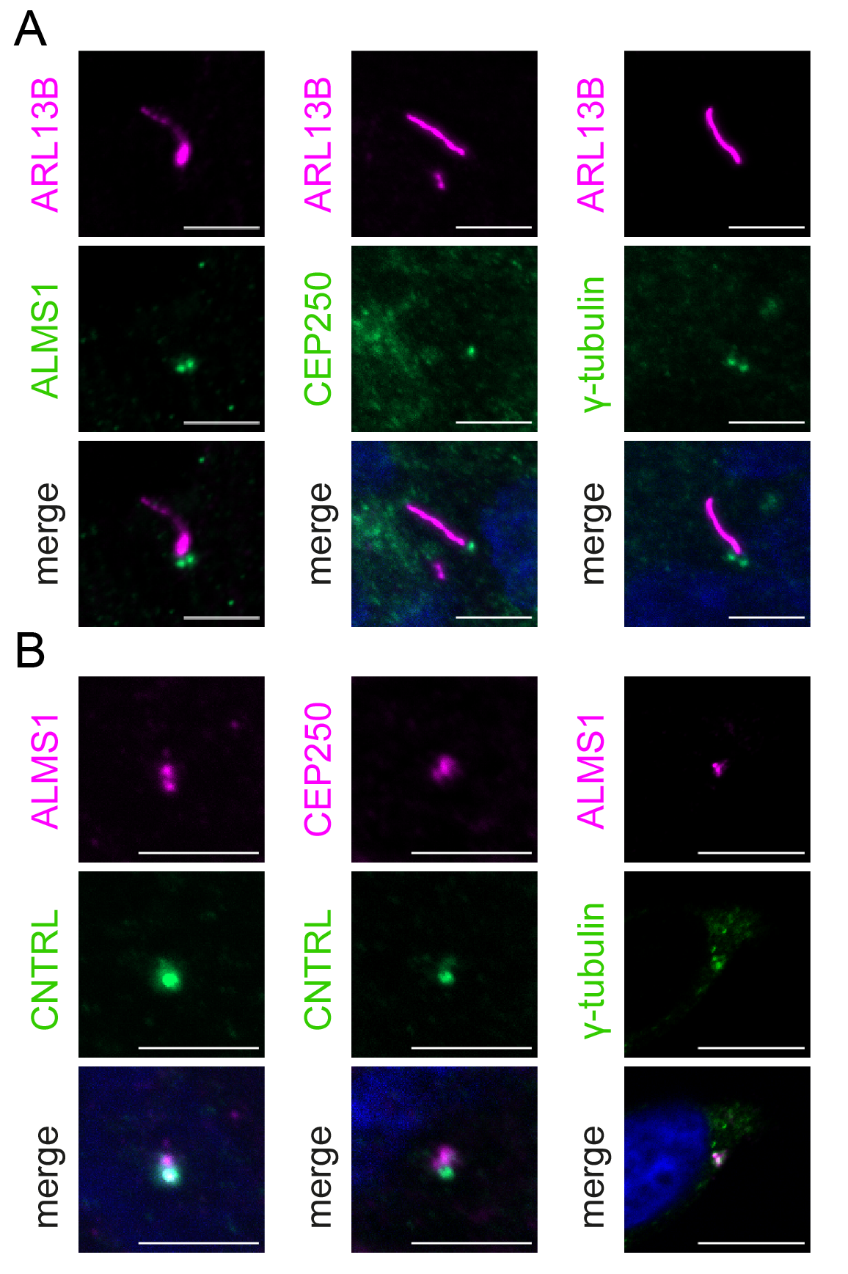
**

Figure S4 Marker detection in untagged HEK293T cells

(A) ALMS1, CEP250 and γ-tubulin (green) were detected at the basal body of cilia (ARL13B, magenta).

(B) Centriolin (CNTRL), γ-tubulin (green) and ALMS1 (magenta) were co-localized in HEK293T wildtype cells. Scale bar = 10µm.


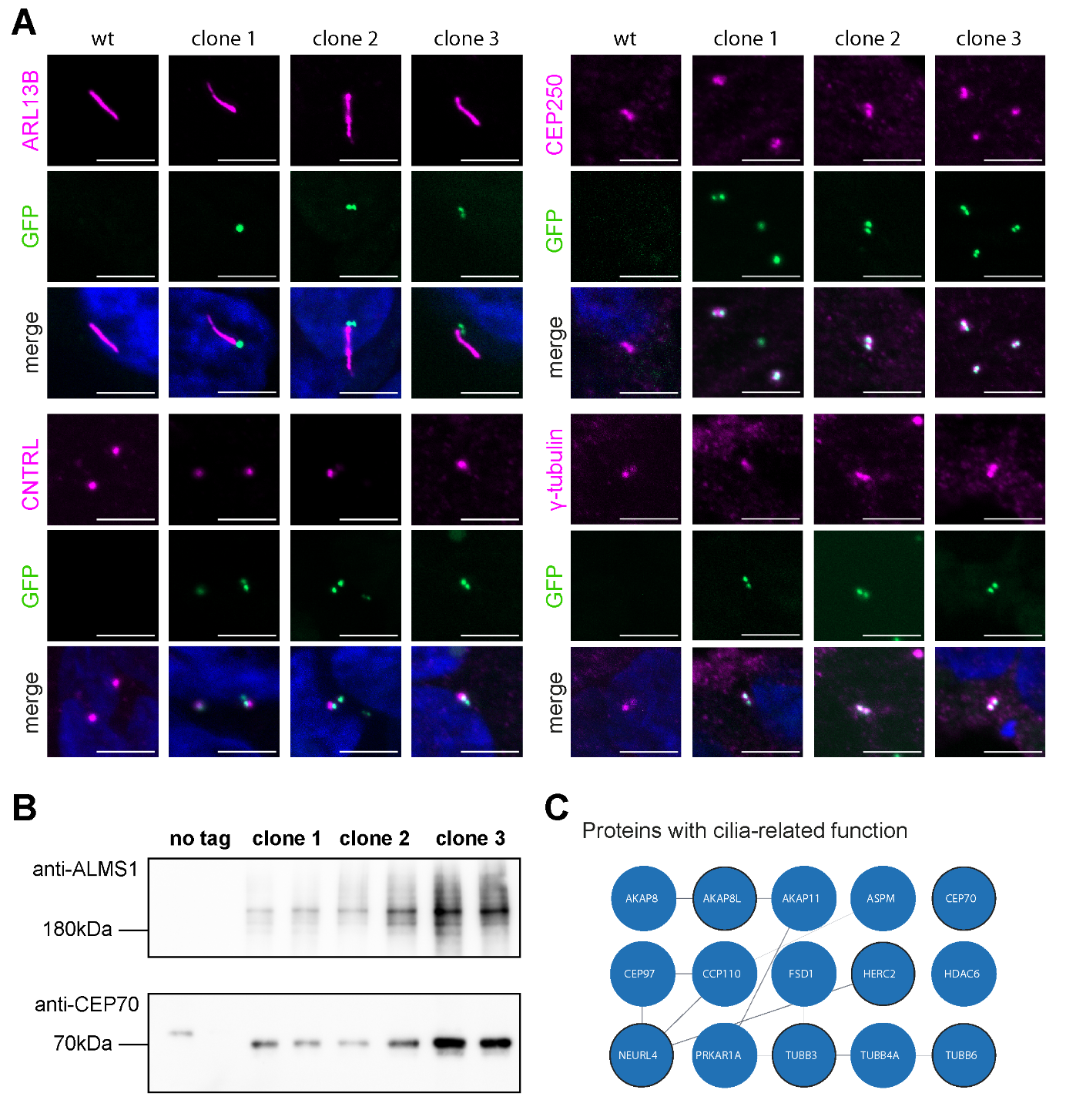


Figure S5 Investigation of 3 ALMS1-sfGFP single clones

(A) Untagged wildtype HEK293T (wt) and three endogenously tagged ALMS1-sfGFP single clones were analyzed for basal body marker localization. In green, endogenously tagged ALMS1 can be detected (GFP) at the base of the cilium (ARL13B, magenta). Normal basal body localization of centriolin (CNTRL), CEP250 and γ-tubulin was shown (magenta). Scale bar = 10µm.

(B) GFP-based affinity purification was performed. Duplicates were investigated by western blot using ALMS1 and CEP70 antibodies. In all three clones an ALMS1 and CEP70 positive band could be detected, with no corresponding band in two untagged control samples.

(C) Mass spectrometry analysis of six biological replicates for each ALMS1-sfGFP clone and the untagged control were analyzed and Tier 2 proteins were determined (Significance A < 0.05, Student’s t-test *p* < 0.05). Tier 2 proteins overlapping in minimum two clones were taken as ALMS1 specific interactors. Here, cilia-related proteins are depicted.

**Table S2 Predicted off-target effects of sgRNA ALMS1 exon 8**

SgRNAs were designed using CCTop and were chosen with low off-target prediction. In this table 20 off-targets, with the highest risk, are depicted. Exons (E) are marked in red, introns (I) in yellow and in (-) green.

| **Coordinates** | **strand** | **MM** | **target_seq** | **PAM** | **distance** | | **gene name** | **gene id** |
| --- | --- | --- | --- | --- | --- | --- | --- | --- |
| [chr2:73453892-73453917](http://genome.ucsc.edu/cgi-bin/hgTracks?db=hg38&position=chr2:73453892-73453917) | + | 0 | TATAACAGATA[GCAGGGAGGAAG] | AGG | 0 | E | ALMS1 | [ENSG00000116127](http://www.ensembl.org/Gene/Summary?g=ENSG00000116127) |
| [chr10:59553566-59553591](http://genome.ucsc.edu/cgi-bin/hgTracks?db=hg38&position=chr10:59553566-59553591) | - | 4 | T**GG**AACAGA**G**A[G**G**AGGGAGGAAG] | AGG | 1688 | - | MRPL50P4 | [ENSG00000235469](http://www.ensembl.org/Gene/Summary?g=ENSG00000235469) |
| [chr9:107375341-107375366](http://genome.ucsc.edu/cgi-bin/hgTracks?db=hg38&position=chr9:107375341-107375366) | + | 4 | TATAA**A**AG**GG**A[GCA**A**GGAGGAAG] | AGG | 43147 | - | RAD23B | [ENSG00000119318](http://www.ensembl.org/Gene/Summary?g=ENSG00000119318) |
| [chrY:12112710-12112735](http://genome.ucsc.edu/cgi-bin/hgTracks?db=hg38&position=chrY:12112710-12112735) | - | 4 | TATAA**A**AGA**A**A[G**GG**GGGAGGAAG] | GGG | NA | - | NA | NA |
| [chr5:71509080-71509105](http://genome.ucsc.edu/cgi-bin/hgTracks?db=hg38&position=chr5:71509080-71509105) | + | 3 | TATAA**A**AGATA[G**A**AGGGA**T**GAAG] | AGG | 360 | I | BDP1 | [ENSG00000145734](http://www.ensembl.org/Gene/Summary?g=ENSG00000145734) |
| [chr9:68759298-68759323](http://genome.ucsc.edu/cgi-bin/hgTracks?db=hg38&position=chr9:68759298-68759323) | + | 4 | TATA**G**CA**T**A**A**A[GCAGGGAG**T**AAG] | GGG | 16641 | I | PIP5K1B | [ENSG00000107242](http://www.ensembl.org/Gene/Summary?g=ENSG00000107242) |
| [chr16:17969552-17969577](http://genome.ucsc.edu/cgi-bin/hgTracks?db=hg38&position=chr16:17969552-17969577) | - | 4 | **G**ATAA**A**AGATA[GCAGG**C**A**T**GAAG] | GGG | 1864 | I | CTA-481E9.4 | [ENSG00000259929](http://www.ensembl.org/Gene/Summary?g=ENSG00000259929) |
| [chr15:29591423-29591448](http://genome.ucsc.edu/cgi-bin/hgTracks?db=hg38&position=chr15:29591423-29591448) | - | 4 | **C**ATAACAG**G**TA[GCAGG**T**AGGA**T**G] | GGG | 18228 | - | RP11-300A12.2 | [ENSG00000259814](http://www.ensembl.org/Gene/Summary?g=ENSG00000259814) |
| [chr2:239006889-239006914](http://genome.ucsc.edu/cgi-bin/hgTracks?db=hg38&position=chr2:239006889-239006914) | + | 4 | TATA**T**CAGA**C**A[GCAGG**A**AGGA**G**G] | AGG | 33879 | - | AC114788.1 | [ENSG00000211566](http://www.ensembl.org/Gene/Summary?g=ENSG00000211566) |
| [chr19:30766564-30766589](http://genome.ucsc.edu/cgi-bin/hgTracks?db=hg38&position=chr19:30766564-30766589) | - | 4 | TATAA**G**AGAT**G**[G**A**AGGGAGGAA**T**] | TGG | 53026 | - | ZNF536 | [ENSG00000198597](http://www.ensembl.org/Gene/Summary?g=ENSG00000198597) |
| [chr8:126681920-126681945](http://genome.ucsc.edu/cgi-bin/hgTracks?db=hg38&position=chr8:126681920-126681945) | + | 4 | TA**A**AACAGA**A**A[GCAGGGAG**C**A**T**G] | TGG | 5507 | I | RP11-89K10.1 | [ENSG00000254286](http://www.ensembl.org/Gene/Summary?g=ENSG00000254286) |

**Table S3 Predicted off-target effects of sgRNA ALMS1 exon 10**

SgRNAs were designed using CCTop and were chosen with low off-target prediction. In this table 20 off-targets, with the highest risk, are depicted. Exons (E) are marked in red, introns (I) in yellow and in (-) green.

| **Coordinates** | **strand** | **MM** | **target_seq** | **PAM** | **distance** | | **gene name** | **gene id** |
| --- | --- | --- | --- | --- | --- | --- | --- | --- |
| [chr2:73490479-73490501](http://genome.ucsc.edu/cgi-bin/hgTracks?db=hg38&position=chr2:73490479-73490501) | + | 0 | TAACCATA[CCCTTATTAGCA] | TGG | 0 | E | ALMS1 | [ENSG00000116127](http://www.ensembl.org/Gene/Summary?g=ENSG00000116127) |
| [chrX:13282113-13282135](http://genome.ucsc.edu/cgi-bin/hgTracks?db=hg38&position=chrX:13282113-13282135) | + | 4 | **A**AA**A**C**C**TA[**A**CCTTATTAGCA] | TGG | 5294 | I | GS1-600G8.5 | [ENSG00000235385](http://www.ensembl.org/Gene/Summary?g=ENSG00000235385) |
| [chr10:65095253-65095275](http://genome.ucsc.edu/cgi-bin/hgTracks?db=hg38&position=chr10:65095253-65095275) | + | 3 | TA**T**CCA**C**A[CC**A**TTATTAGCA] | TGG | 28392 | - | RP11-252C24.3 | [ENSG00000282906](http://www.ensembl.org/Gene/Summary?g=ENSG00000282906) |
| [chr12:63854927-63854949](http://genome.ucsc.edu/cgi-bin/hgTracks?db=hg38&position=chr12:63854927-63854949) | + | 4 | **A**AACC**T**T**T**[C**T**CTTATTAGCA] | GGG | 10044 | I | SRGAP1 | [ENSG00000196935](http://www.ensembl.org/Gene/Summary?g=ENSG00000196935) |
| [chrX:53352221-53352243](http://genome.ucsc.edu/cgi-bin/hgTracks?db=hg38&position=chrX:53352221-53352243) | + | 4 | **CC**ACCA**C**A[CCC**C**TATTAGCA] | TGG | 10716 | - | RP6-29D12.2 | [ENSG00000234413](http://www.ensembl.org/Gene/Summary?g=ENSG00000234413) |
| [chr2:105343534-105343556](http://genome.ucsc.edu/cgi-bin/hgTracks?db=hg38&position=chr2:105343534-105343556) | + | 4 | TAA**AT**AT**T**[CC**T**TTATTAGCA] | AGG | 311 | I | C2orf49 | [ENSG00000135974](http://www.ensembl.org/Gene/Summary?g=ENSG00000135974) |
| [chrX:111675757-111675779](http://genome.ucsc.edu/cgi-bin/hgTracks?db=hg38&position=chrX:111675757-111675779) | - | 4 | T**TC**CCATA[C**AT**TTATTAGCA] | AGG | 5339 | I | ALG13 | [ENSG00000101901](http://www.ensembl.org/Gene/Summary?g=ENSG00000101901) |
| [chr8:137454746-137454768](http://genome.ucsc.edu/cgi-bin/hgTracks?db=hg38&position=chr8:137454746-137454768) | + | 4 | **G**AA**T**CATA[C**AT**TTATTAGCA] | TGG | 29725 | - | ZYXP1 | [ENSG00000274572](http://www.ensembl.org/Gene/Summary?g=ENSG00000274572) |
| [chr14:23710784-23710806](http://genome.ucsc.edu/cgi-bin/hgTracks?db=hg38&position=chr14:23710784-23710806) | + | 4 | T**TT**C**T**ATA[CCCTT**C**TTAGCA] | TGG | 18232 | - | RP11-388E23.2 | [ENSG00000258464](http://www.ensembl.org/Gene/Summary?g=ENSG00000258464) |
| [chr16:8613848-8613870](http://genome.ucsc.edu/cgi-bin/hgTracks?db=hg38&position=chr16:8613848-8613870) | + | 4 | T**C**AC**T**ATA[C**A**C**C**TATTAGCA] | TGG | 7813 | - | METTL22 | [ENSG00000067365](http://www.ensembl.org/Gene/Summary?g=ENSG00000067365) |
| [chr10:16045742-16045764](http://genome.ucsc.edu/cgi-bin/hgTracks?db=hg38&position=chr10:16045742-16045764) | - | 4 | T**T**ACCA**C**A[C**A**C**C**TATTAGCA] | TGG | 25652 | - | FTLP19 | [ENSG00000237913](http://www.ensembl.org/Gene/Summary?g=ENSG00000237913) |
| [chr2:99859286-99859308](http://genome.ucsc.edu/cgi-bin/hgTracks?db=hg38&position=chr2:99859286-99859308) | + | 4 | **G**AACCA**C**A[**T**CCTT**T**TTAGCA] | TGG | 21762 | I | AFF3 | [ENSG00000144218](http://www.ensembl.org/Gene/Summary?g=ENSG00000144218) |
| [chr5:78044294-78044316](http://genome.ucsc.edu/cgi-bin/hgTracks?db=hg38&position=chr5:78044294-78044316) | - | 4 | T**T**A**T**CATA[**G**CCTTA**G**TAGCA] | TGG | 156 | I | CTD-2179L22.1 | [ENSG00000253558](http://www.ensembl.org/Gene/Summary?g=ENSG00000253558) |
| [chr2:59186123-59186145](http://genome.ucsc.edu/cgi-bin/hgTracks?db=hg38&position=chr2:59186123-59186145) | + | 3 | **G**AACCATA[CCCT**G**A**G**TAGCA] | AGG | 31563 | - | AC007131.1 | [ENSG00000222030](http://www.ensembl.org/Gene/Summary?g=ENSG00000222030) |
| [chr6:130076432-130076454](http://genome.ucsc.edu/cgi-bin/hgTracks?db=hg38&position=chr6:130076432-130076454) | + | 4 | TAAC**TT**TA[**T**CCTTAT**A**AGCA] | TGG | 2104 | I | L3MBTL3 | [ENSG00000198945](http://www.ensembl.org/Gene/Summary?g=ENSG00000198945) |
| [chr19:47805134-47805156](http://genome.ucsc.edu/cgi-bin/hgTracks?db=hg38&position=chr19:47805134-47805156) | - | 4 | **A**AACCA**A**A[CC**A**TTAT**C**AGCA] | GGG | 1445 | I | TPRX1 | [ENSG00000178928](http://www.ensembl.org/Gene/Summary?g=ENSG00000178928) |
| [chr1:61159505-61159527](http://genome.ucsc.edu/cgi-bin/hgTracks?db=hg38&position=chr1:61159505-61159527) | + | 4 | T**G**ACCA**C**A[CCCT**G**A**G**TAGCA] | AGG | 26477 | I | NFIA | [ENSG00000162599](http://www.ensembl.org/Gene/Summary?g=ENSG00000162599) |
| [chr6:8376736-8376758](http://genome.ucsc.edu/cgi-bin/hgTracks?db=hg38&position=chr6:8376736-8376758) | - | 4 | T**G**ACCA**C**A[CCCT**G**A**G**TAGCA] | AGG | 33715 | - | RP11-203H2.2 | [ENSG00000234763](http://www.ensembl.org/Gene/Summary?g=ENSG00000234763) |
| [chr1:4427563-4427585](http://genome.ucsc.edu/cgi-bin/hgTracks?db=hg38&position=chr1:4427563-4427585) | - | 4 | TAAC**T**A**C**A[CCCT**G**A**C**TAGCA] | AGG | 2874 | - | RP5-1166F10.1 | [ENSG00000235054](http://www.ensembl.org/Gene/Summary?g=ENSG00000235054) |
| [chr8:74307346-74307368](http://genome.ucsc.edu/cgi-bin/hgTracks?db=hg38&position=chr8:74307346-74307368) | - | 4 | T**T**ACC**T**TA[CCCTT**CA**TAGCA] | GGG | 7493 | I | JPH1 | [ENSG00000104369](http://www.ensembl.org/Gene/Summary?g=ENSG00000104369) |


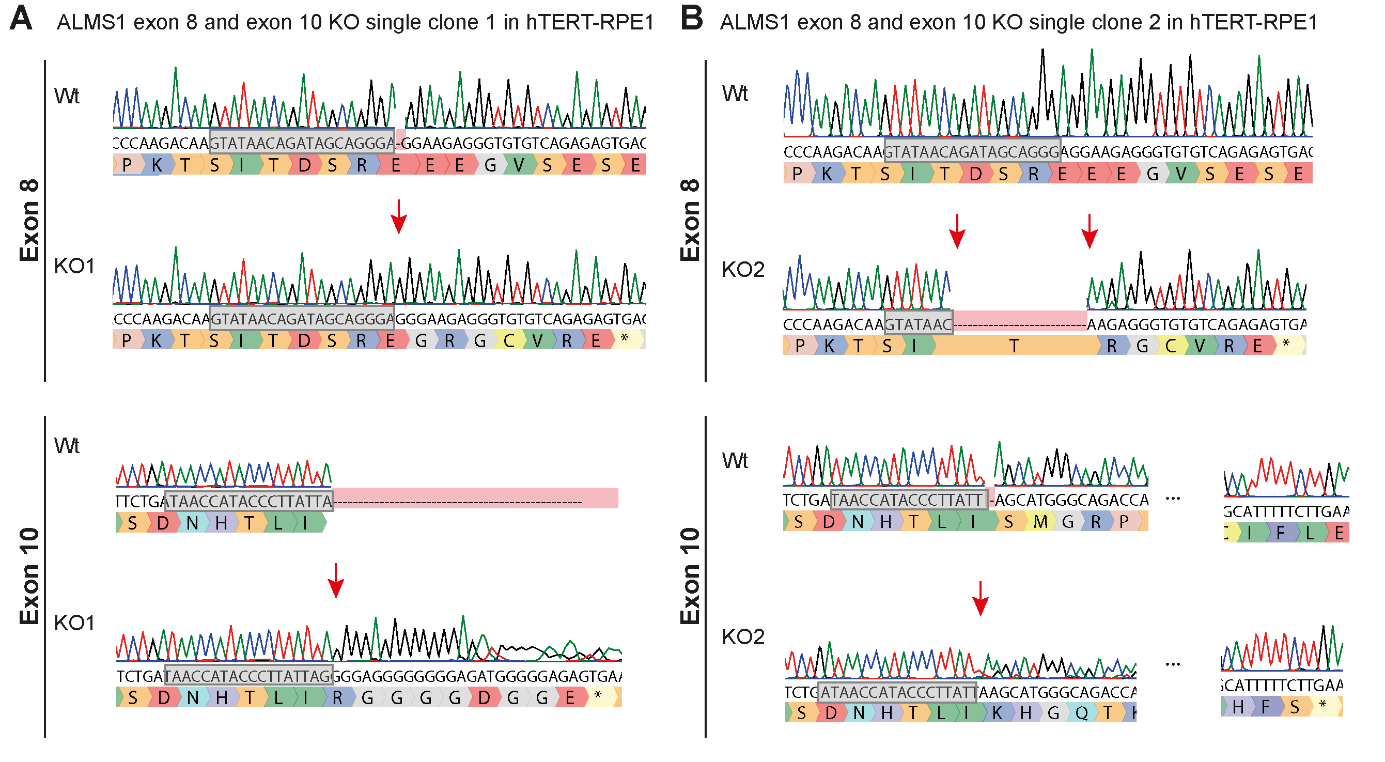


Figure S6 Verification of CRISPR/Cas9 induced indels in hTERT-RPE1 cells

Electropherograms of CRISPR/Cas9 mediated ALMS1 KO in hTERT-RPE1 single clones with their respective nucleotide and amino acid sequences are depicted. SgRNAs are depicted in grey. Overview was created with Benchling [Biology Software], 2023, retrieved from <https://benchling.com>. The introduced indels exhibit frameshift mutations that lead to an early stop codon.

(A) ALMS1 KO1 with indels in exon 8 (p.Glu2462Glyfs*8) and exon 10 (p.Ser2846Argfs*10). On the top panel the native ALMS1 exon 8 sequence with the marked indel region (red arrow) is shown, followed by the KO sequence with a G insertion, leading to an early stop codon (*). The same is depicted for ALMS1 exon 10.

(B) ALMS1 KO2 with indels in exon 8 (p.Asp2458Argfs*7) and exon 10 (p.Ser2846Lysfs*38).


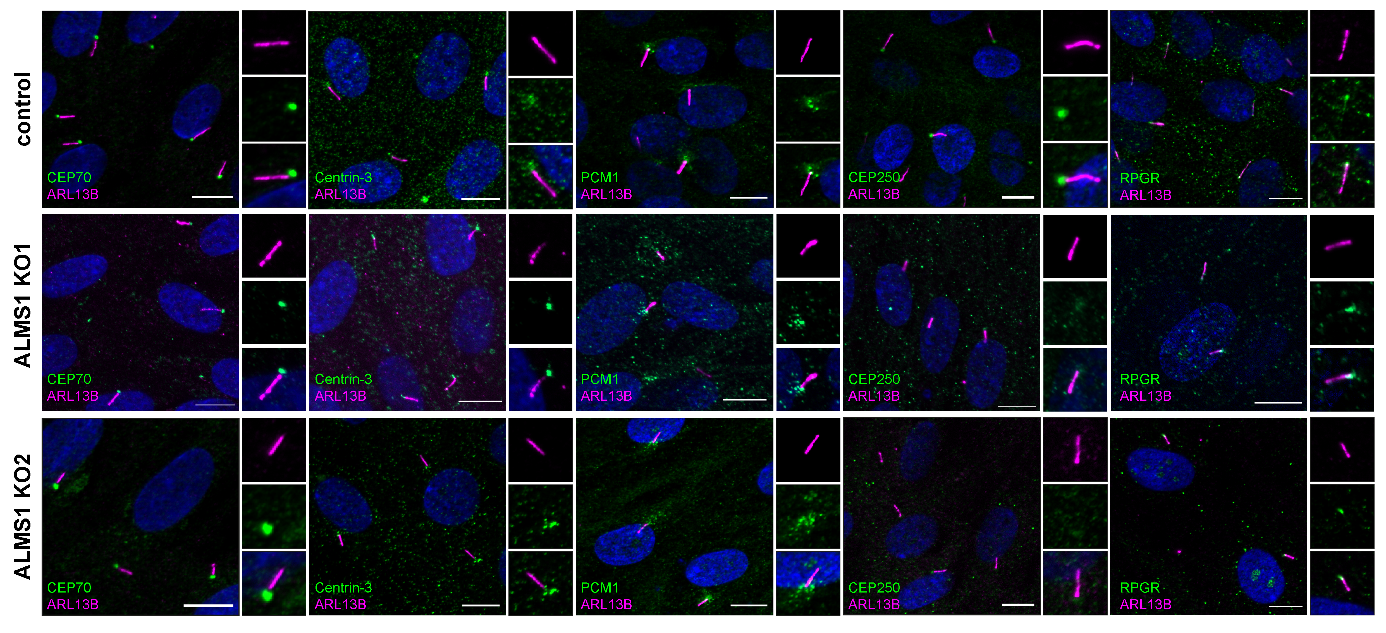


Figure S7 Ciliary protein localization in ALMS1 KO cells

ALMS1 KO cells and control cells were co-stained for cilia (ARL13B, magenta) and CEP70, Centrin-3, PCM1, CEP250 or RPGR (green), respectively. No changes were seen for most markers, whereas CEP250 was reduced at the BB, as described before. Scale bar = 10µm


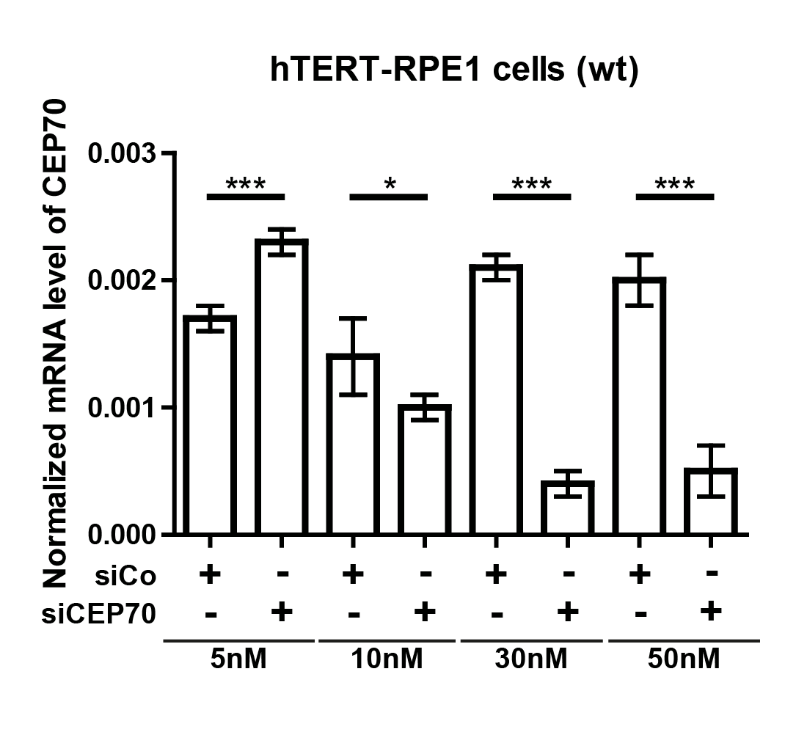


Figure S8 Knock-down of CEP70 in hTERT-RPE1 wildtype cells

Knock-down with a pool of three CEP70 siRNAs (siCEP70) with different concentrations ranging from 5-50nM was conducted. CEP70 qPCR primer spam from exon 9 to 11. GAPDH was used as housekeeping gene for normalization. SiControl = SiCo, + = with, - = without.

**
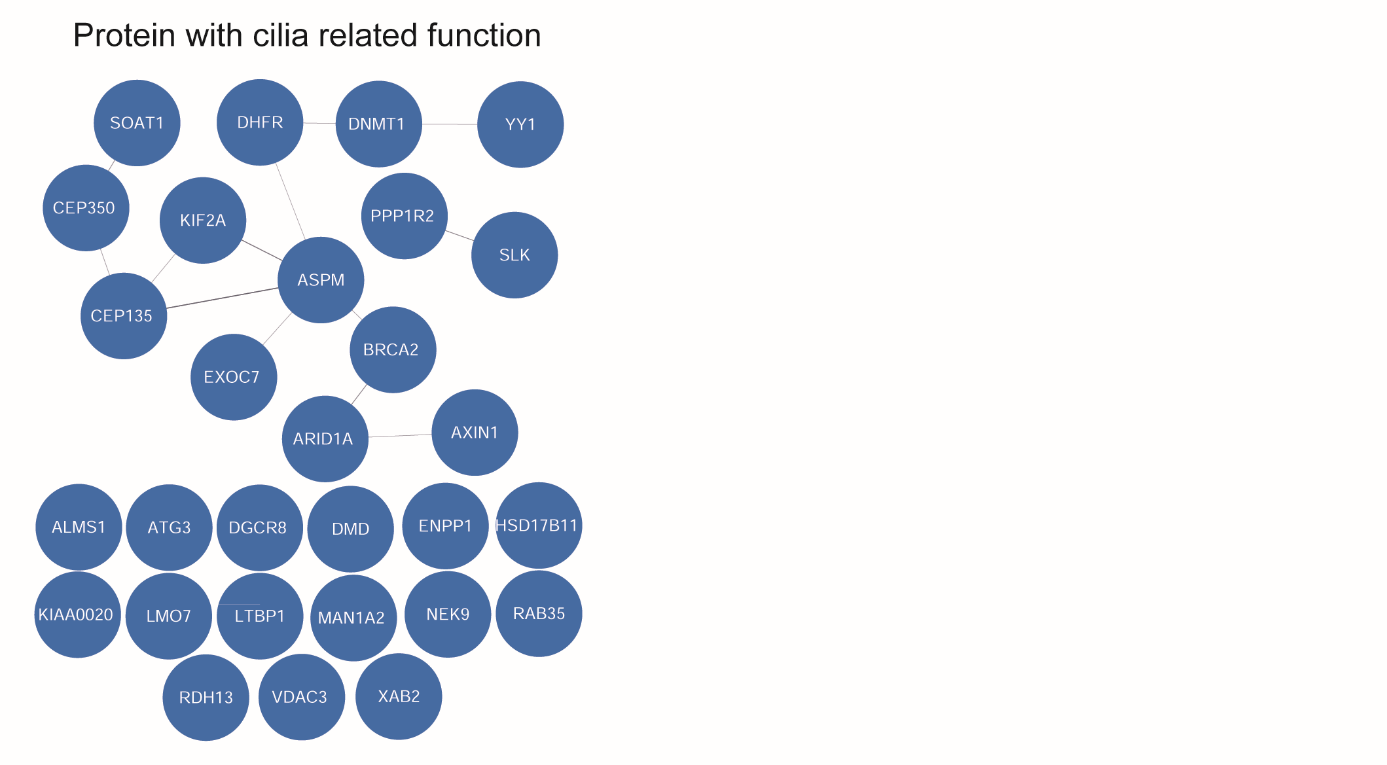
**

**Figure S9 CEP70 interactors play a role in cilia-related function**

Tier 2 proteins (Significance A <0.05 (Benjamini-Hochberg <0.05) and *p*-value < 0.05), that play a role in cilia biology are depicted in blue.


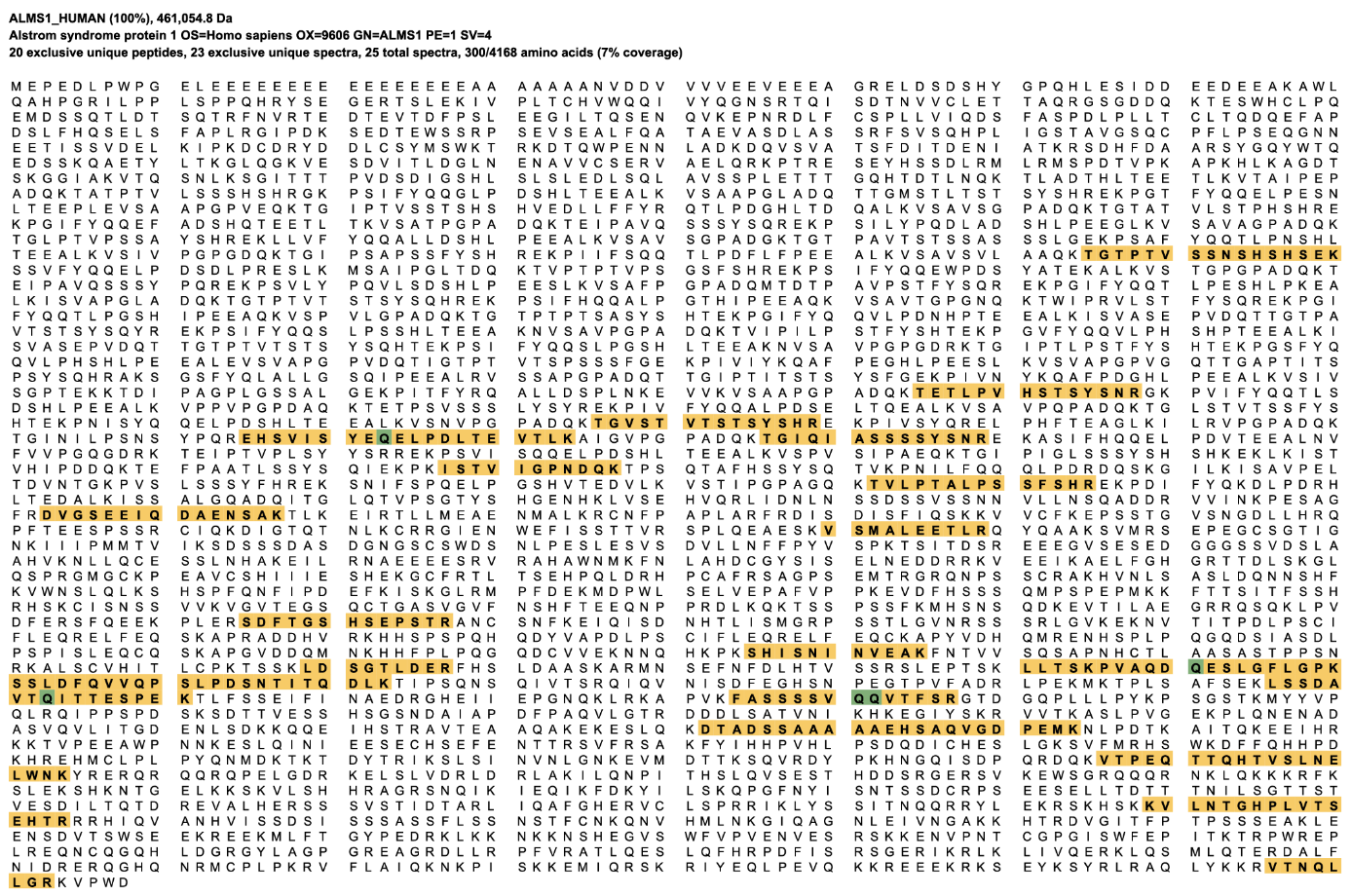


Figure S10 ALMS1 coverage in (N)Strep/FLAG-CEP70 sample

Localization of identified peptides (exclusive unique peptides) of the ALMS1 protein (homo sapiens, 461 kDa) in an CEP70 NSF sample provided by the program Scaffold is depicted. Amino acids matched to MS/MS spectrum are marked in yellow. Amino acids with a post-translational modification are shown in green.

**Primer List**

| ALMS1_exon8 forward | GCAGAGAGCAAAGTCAGTATG |
| --- | --- |
| ALMS1_exon8 reverse | TTTCATTGGCTAAGCTTCCTC |
| ALMS1_exon10 forward | ACCCTTGGCTGTCAGAATTAG |
| ALMS1_exon10 reverse | GATGTTGAGGAGAGGGAGAATG |
| ALMS1_exon23 forward | CAGATCCTCTTTCCTGAACCTTTCG |
| ALMS1_exon23 reverse | CCCTCATTGGTGAGTCAGATA |

**Oligonucleotide for sgRNA cloning List**

| ALMS1 sgRNA exon8 bottom | AAACCTTCCTCCCTGCTATCTGTTATAC |
| --- | --- |
| ALMS1 sgRNA exon8 top | CACCGTATAACAGATAGCAGGGAGGAAG |
| ALMS1 sgRNA exon10 bottom | CACCGTAACCATACCCTTATTAGCA |
| ALMS1 sgRNA exon10_top | AAACTGCTAATAAGGGTATGGTTAC |
| ALMS1 sgRNA exon23 bottom | AAACAGGGAACTTTTCTCCCCAGAC |
| ALMS1 sgRNA exon23 top | CACCGTCTGGGGAGAAAAGTTCCCT |

**QPCR Primer List**

| Primer name | 5’-3’ direction |
| --- | --- |
| CEP70 exon 3 forward | GGATTCCAGTCAACCATCAG |
| CEP70 exon 5 reverse | CAAATTCTGTCTCATCCTTTGTG |
| CEP70 exon 9 forward | CAGAAATCTGGATGCCTCAC |
| CEP70 exon 11 reverse | GCTTCTTCACCTGCTGTTTA |
| GAPDH forward | GCAAATTCCATGGCACCGT |
| GAPDH reverse | GCCCCACTTGATTTTGGAGG |

**qPCR Setting (Two step PCR with melting curve)**

| 95 °C | 1 min | |
| --- | --- | --- |
| 95 °C | 10 s | 40 cycles |
| 60 °C | 30 s |  |
| 65 °C | 5 s, 0.5 °C/cycle | |
| 95 °C | 5 s, 0.5 °C/cycle | |

**Abbreviations**

| % | Percentage |
| --- | --- |
| µg | Micrograms |
| µl | Microliter |
| µm | Micrometer |
| aa | Amino acid |
| bp | Base pair |
| BSA | Bovine Serum Albumin |
| CEP70 | Centrosomal protein 70kDa |
| CRISPR/Cas9 | clustered regularly interspaced short palindromic repeats/CRISPR-associated nuclease 9 |
| CT | C-Terminal |
| DAPI | 4’,6-Diamidin-2-phenylindol |
| DMEM | Dulbecco’s Modified Eagle Medium |
| dNTPS | Desoxynuceloside-Triphosphate |
| DPBS | Dulbecco's Phosphate-Buffered Saline |
| DS NC1 | Nontargeting DsiRNA |
| DsiRNA | Dicer-substrate siRNA |
| E.Coli | Escherichia coli |
| g | Gram |
| h | hour |
| HEK293T | human embryonic kidney cells |
| HPLC | high performance liquid chromatography |
| hTERT-RPE1 | human telomerase reverse transcriptase immortalized retina pigmented epithelial cells |
| Kb | Kilobases |
| KD | Knockdown |
| kDa | Kilo Dalton |
| KI | Knock-in |
| KO | Knockout |
| LB | Lysis buffer |
| LC | Liquid chromatography |
| LC-MS/MS | Liquid chromatography coupled to mass spectrometry |
| MDa | Megadalton |
| min | Minute |
| MS | Mass spectrometry |
| MTOC | Microtubule organizing center |
| NGS | Normal goat serum |
| NGS | Normal goat serum |
| nM | Millimolar |
| NP40 | Nonidet-P40 |
| NSF | N-terminal Strep Flag |
| NT | N-terminal |
| PBST | Dulbecco’s Phosphate-Buffered Saline mit Triton |
| PCR | Polymerase Chain Reaction |
| PEI | Polyethylenimine linear |
| PFA | Paraformaldehyde |
| PI 2/3 | Phosphotase Inhibitor Cocktail |
| PIC | Protease inhibitor complex complete |
| qPCR | Quantitative PCR |
| SD | Standard deviation |
| sfGFP | Super folder green fluorescence protein |
| sgRNA | Single guide RNA |
| DsiRNA | Dicer-substrate short interfering RNA |
| TFA | Trifluoracetic Acid |
| wt | Wildtype |
| x g (rcf) | Relative Centrifugal Force |
