# Supplementary material for: Interactome Analysis Reveals a Link of the Novel ALMS1-CEP70 Complex to Centrosomal Clusters
Source: Mol Cell Proteomics. 2023 Dec 18;23(1):100701. doi: 10.1016/j.mcpro.2023.100701 (PMC10820798; doi:10.1016/j.mcpro.2023.100701)
Supplement: Supplementary table legends [file mmc2.docx]

**Supplementary table legends**

Table S4 ALMS1 interaction data

ALMS1-sfGFP Tier 1 and Tier 2 significantly enriched proteins, purified by performing GFP-based affinity purification for three HEK293T single clones versus untagged control, are shown (log2 ratio of median tagged clone/untagged control based on 6 biological replicates each; Tier 1: Significance A (Benjamini Hochberg) < 0.05; Student's t-test (Permutation based FDR) < 0.05; Tier 2: Significance A (Benjamini Hochberg) < 0.05; Student's t-test (*p*-value) < 0.05)). ALMS1 is depicted in green. Tier 2 proteins which overlapped in minimum two clones were defined as ALMS1 network proteins. For the VENN diagram, the following free online tool was used: <https://bioinformatics.psb.ugent.be/webtools/Venn/>.

Table S5 GO enrichment analysis ALMS1

GO enrichment of ALMS1 network proteins was done using the GO Ontology database. Proteins localized to spindle, centrosome or microtubule organizing center (MTOC), which might help to understand ALMS1-related functions, are shown.

Table S6 CEP70 interaction data

Full length Strep/FLAG-tagged CEP70 was transfected into HEK293T cells and affinity purification was performed. Significantly enriched proteins in CEP70 samples were determined (Tier 1: Significance A (Benjamini Hochberg) < 0.05; Student's t-test (Permutation based FDR) < 0.05; Tier 2: Significance A (Benjamini Hochberg) < 0.05; Student's t-test (*p*-value) < 0.05). In parallel, deletion fragments expressing either the coiled-coil (CC1-2) or the TPR CEP70 domain, respectively, were investigated. Ciliary proteins which were reduced or lost with just expressing the CC1-2 domain compared to the full-length CEP70 are depicted in red.

Table S7 GO enrichment analysis CEP70

GO enrichment of CEP70 interacting proteins was done using the GO Ontology database.

Table S8 MaxQuant parameters ALMS1-sfGFP endogenously tagged clones

Table S9 MaxQuant parameters CEP70 full length and fragment analysis
